# A First Insight into the Genome of the Filter-Feeder Mussel *Mytilus galloprovincialis*

**DOI:** 10.1371/journal.pone.0151561

**Published:** 2016-03-15

**Authors:** Maria Murgarella, Daniela Puiu, Beatriz Novoa, Antonio Figueras, David Posada, Carlos Canchaya

**Affiliations:** 1 Department of Biochemistry, Genetics and Immunology and Unidad Asociada CSIC, University of Vigo, Vigo, Spain; 2 Center for Computational Biology. McKusick-Nathans, Institute of Genetic Medicine, Johns Hopkins University School of Medicine, Baltimore, Maryland, United States of America; 3 Instituto de Investigaciones Marinas, Consejo Superior de Investigaciones Científicas, Vigo, Spain; Glasgow Caledonian University, UNITED KINGDOM

## Abstract

Mussels belong to the phylum Mollusca, one of the largest and most diverse taxa in the animal kingdom. Despite their importance in aquaculture and in biology in general, genomic resources from mussels are still scarce. To broaden and increase the genomic knowledge in this family, we carried out a whole-genome sequencing study of the cosmopolitan Mediterranean mussel (*Mytilus galloprovincialis*). We sequenced its genome (32X depth of coverage) on the Illumina platform using three pair-end libraries with different insert sizes. The large number of contigs obtained pointed out a highly complex genome of 1.6 Gb where repeated elements seem to be widespread (~30% of the genome), a feature that is also shared with other marine molluscs. Notwithstanding the limitations of our genome sequencing, we were able to reconstruct two mitochondrial genomes and predict 10,891 putative genes. A comparative analysis with other molluscs revealed a gene enrichment of gene ontology categories related to multixenobiotic resistance, glutamate biosynthetic process, and the maintenance of ciliary structures.

## Introduction

Mussels belong to the phylum Mollusca, one of the largest and most diverse taxa in the animal kingdom, only second to Insecta. The number of molluscan species has been estimated to be 93,000 [1], with 25% of them being marine. Among all classes belonging to this phylum, bivalves–where mussels belong–show the most highly modified body plan, flattened side-to-side, over evolutionary time. Morphologically, they are characterized by the presence of a bivalve shell, filtrating gills, no differentiated head, and a lack of radula. Other anatomical features such as adult byssal attachment and mantle fusion may have also played an important role in their adaptation as filter feeders and burrowers, respectively [2]. Some bivalves show an atypical double uniparental inheritance (DUI) of mitochondria. In these species, all progeny inherit one mitochondrial genome from the mother (F-type), while males also receive a mitochondrial genome from their father (M-type). This DUI, initially described in *M*. *edulis* [3], has been extensively studied in the genus *Mytilus* [4,5]. Another remarkable characteristic of mussels is their natural resistance to diseases. Unlike vertebrates, that have also developed an adaptive immune system, the immune system of bivalves is solely based on innate defences, which play a prominent role in protecting these animals against invading microorganisms. However, differences in disease resistance have been observed among bivalves. Compared to other edible bivalves, like oysters and clams, mussels seem far less susceptible to mass mortalities and diseases [6,7]. Interestingly, previous mass mortalities in mussels have not been linked to any micro-organisms [8], suggesting that the innate immune system in mussels is highly efficient.

Marine mussels have also a significant commercial value. Their production corresponds to 50% of global EU aquaculture in weight and about 30% in value [9]. In this regard, the most important mussel species are the Mediterranean (*Mytilus galloprovincialis*, Lamarck 1819) and the blue (*Mytilus edulis)* mussels. In Spain, the 2012 farmed production of *M*. *galloprovincialis* was 231,754 TM [10], of which 227,229 TM were produced in Galicia, NW Spain [11]. Indeed, the relevance of its farming in Galicia is not only economical but also social: mussel culture employs about 15,000 people in 2,400 familiar enterprises [9].

Despite the commercial and scientific interest in mussels in biology and aquaculture, the number of genomic resources available in public databases for these organisms is quite limited, and usually restricted to their transcriptomes. For instance, in *M*. *galloprovincialis*, some transcriptome studies using ESTs [12–14] and high throughput cDNA [15,16] are available. Clearly, molecular tools still need to be developed for the management, molecular breeding and genetic manipulation of *Mytilus* spp. in aquaculture [17].

We present here the first genome sequencing study in this genus, namely a low-coverage whole-genome study of the Mediterranean mussel *M*. *galloprovincialis*. Indeed, low-coverage sequencing of non-model organisms can provide valuable information about their genomes [18,19] regarding important features such as gene content, functional elements and repetitive sequences [20–22]. Here, we offer a first insight into the general features and complexity of the genome of *M*. *galloprovincialis*, providing a starting point for future genomic research on this important bivalve. Moreover, the availability of this genome sequence together with those of four other molluscs already sequenced (the California sea hare *Aplysia californica*, the owl limpet *Lottia gigantea* [23], the pacific oyster *Crassostrea gigas* [24] and the pearl oyster *Pinctada fucata* [25]) should improve the knowledge of this important phylum through genomic comparisons at multiple levels.

## Materials and Methods

### Sequencing, k-mer analyses, and assembly

For sequencing, we extracted 4μg of DNA from muscle tissue from a single mussel extracted from the Ria of Vigo, Spain. Using this DNA, three sequencing libraries with insert sizes of 180, 500 and 800 bp were constructed and sequenced at BGI (Beijing Genomics Institute—China). These libraries were sequenced with the Illumina HiSeq2000 high-throughput platform using paired-end sequencing (100-bp reads). To clean the initial set of reads, we filtered out raw reads if they fulfilled any of these conditions: a) >5% ambiguous bases (represented by the letter N); b) poly-A structures; c) > = 20 bases with low quality scores; d) adapter contamination: reads with more than 10 bp aligned to the adapter sequence (no more than 3-bp mismatch allowed); or e) small insert-size reads in which paired reads overlapped more than or equal to 10 bp (10% mismatch allowed).

We used Jellyfish [26] for counting k-mers and obtaining their frequency distributions. With these data, we drew frequency plots using k-mer lengths of 15, 17, 19 and 21. To assign the “true” coverage peak, we compared these plots to identify the peak that changed in height (“heterozygous peak”) and the one that did not (“coverage peak”). The latter was then used to calculate the genome size as the total k-mer number divided by the coverage-peak depth [27]. Finally, we assembled *de novo* the reads resulting from the quality filtering step using SOAPdenovo v1.05 [27] with parameters -*K 31 -d 1 -M 1 -F–R*. Then, we ran the Assemblathon 2 script [28] to obtain assembly statistics. Using this script, we compared the genome assemblies of *M*. *galloprovincialis* with those of *A*. *californica*, *L*. *gigantea*, *P*. *fucata*, and *C*. *gigas* (S1 File). Genome surveys of other molluscs with scarce sequencing depth [22] were not included in these comparisons. We confirmed the identification of the studied mussel as *M*. *galloprovincialis* by scanning the assembled sequences with two *Mytilus* genetic markers, *Glu-5’* [29] and *EFbis* [30], using BLASTN [31] and Geneious version 6.1.8 [32].

### Isolation of mitochondrial sequences and variant calling

For the mitochondrial genome analysis, first we detected contigs in our assembly that matched *M*. *galloprovincialis* mitochondrial DNA sequences. For this, we aligned our contigs against F (GenBank NC_006886, MgF) and M (GenBank AY363687, MgM) *M*. *galloprovincialis* mitochondrial sequences using BLASTN and *nucmer* (from MUMMer version 3.23 [33]). We filtered out BLASTN alignments with e-value above 1x10^-6^, identity < 90%, or contig alignment coverage < 90% to remove non-specific alignments. The program *nucmer* was used with arguments -*maxmatch* and *-c 100*. We mapped the contigs that aligned (using *nucmer*) with MgF and constructed its corresponding tiling path (S1 Fig). For both BLAST and *nucmer*, we also calculated the proportion of MgF and MgM nucleotides that aligned with the assembly. Second, for variant calling, we mapped the cleaned reads against several mitochondrial genomes of *Mytilus* spp. (S1 File) using Bowtie2 v2.0.6 [34] with the option “very-sensitive”, identifying single-nucleotide variants (SNVs) with SAMtools version 0.1.18 [35].

### Repeat Sequence Analysis

To estimate the amount and composition of repetitive elements in our assembled genome, we carried out three different analyses using RepeatMasker (http://www.repeatmasker.org). First, we ran RepeatMasker against our working assembly using the default repeat sequence entries of the genus *Mytilus*. For this, we used the Repbase database [36] version 20120418 (we called this search “MYTILUS”). To overcome the limitation of MYTILUS due to the low number of *M*. *galloprovincialis* entries present in Repbase, a second analysis was performed using the Repbase entries from the phylum Mollusca (search “MOLLUSCA”). Finally, a third RepeatMasker analysis (search “INHOUSE”) was carried out using our own in-house library of repetitive elements. This library was built from putative repetitive sequences obtained using RepeatModeler (http://www.repeatmasker.org/RepeatModeler.html). We repeated similar analyses with the other four molluscan genomes studied (S1 File).

### Gene Prediction and Annotation

Before starting the gene prediction and annotation steps, we searched for potential contaminant sequences in our assembly. We looked for sequences with bacterial signatures using Kraken 0.10.5-beta [37] and BLASTX. First, we selected contigs with bacterial k-mer matches using Kraken. Second, we calculated the accumulated length of the matched regions in each of these contigs. Third, we putatively annotated as “bacterial” those contigs whose aligned regions added up more than 10% of the total contig length. Fourth, we confirmed the putative bacterial origin by matching the resulting contigs using BLASTX against the non-redundant (nr) database with a cut-off e-value of 1.0x10^-6^. Finally, we annotated as bacterial those resulting contigs that best matched proteins of bacterial origin. We did not include these putative bacterial sequences in the subsequent analyses.

Next, to estimate the quality of the predicted mussel gene repertoire, we used the program CEGMA [38] with default options. This tool quantifies the completeness of a gene repertoire within a genome assembly by aligning its sequences to a "universal" set of 248 eukaryotic core proteins. For CEGMA, significant sequence alignments spanning more than 90% of the length of any CEGMA protein are classified as being "complete" in the analysed set of sequences. To estimate the gene repertoire size in *M*. *galloprovincialis*, we assigned the percentage of CEGMA sequences found in our assembly as the percentage of the *M*. *galloprovincialis* gene repertoire in our assembly [38].

We used MAKER2 [39] to predict *in silico* the gene models in our assembly. Additional *M*. *galloprovincialis* experimental sequence data coming from two other different sources were used to improve the gene prediction with MAKER2. These data consisted of our in-house RNA-Seq *de novo* assemblies from 4 different tissues, and protein sequences from the NCBI database. We used these 2 different datasets to build a first *ab initio* gene prediction model using the program SNAP [40]. Finally, MAKER2 was used with the resulting previous SNAP results to accomplish a refined, more reliable gene prediction. To characterize and validate the resulting protein sequences, we aligned the contigs with mpiBLAST [41] against the *nr* database (e-value cut-off of 1 x 10^−6^). Finally, we used Blast2GO [42] with the BLAST *nr* database and InterProScan 5RC7 [43] to obtain a more complete functional protein annotation and description of our genome survey. Using this bioinformatic tool, we also compared the functional protein annotation of *M*. *galloprovincialis* with those of the other four molluscs (S1 File). The comparison was done using the Fisher's exact test, with a False Discovery Rate (FDR) of 0.05. We included only those ontologies that belonged to the “biological function” category and presented significant differences with other molluscan datasets.

## Results and Discussion

### Genome sequence composition and size

**Fig 1:**
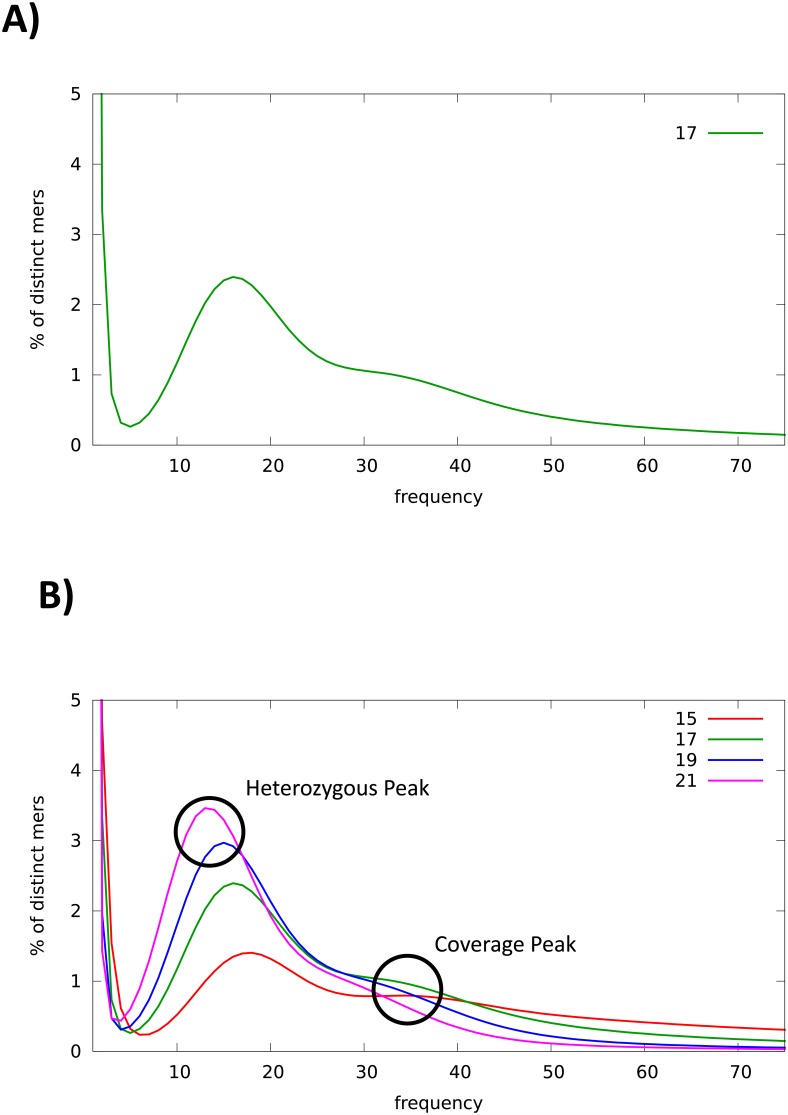
**A)** Histogram of 17 k-mers. **B)** K-mer frequency distributions ad different k-mer lengths.

The obtained k-mer frequency plots for *M*. *galloprovincialis* were bimodal, with two clear peaks at 16X and 32X (Fig 1A; 17-mers). A pattern like this has been previously reported for other genomes [44,45], and in simulations [46], and it is thought to be a direct consequence of heterozygosity in diploid genomes. To identify which of these two peaks was the coverage peak, we generated several k-mer frequency distributions with different k-mer sizes (Fig 1B). We observed that the peak height at 16X changed considerably for different k-mer sizes, while the peak height at 32X remained more or less stable. Therefore, we concluded that the latter peak was the coverage peak, containing k-mers from homozygous regions, while the former was the heterozygous peak, containing k-mers from heterozygous regions. The observed sharp difference in height between the homozygous and heterozygous peaks (Fig 1B; e.g. 21-mer plot) may be produced by a seemingly high heterozygosity within the *M*. *galloprovincialis* genome. This is not surprising, as species within the genus *Mytilus* have been previously reported to have high genetic diversity [47–51]. In a genomic survey [52] of 76 non-model organisms using RNA-Seq data, *M*. *trossulus* occupied the second highest value of genetic diversity while *M*. *galloprovinci*alis occupied the 15^th^.

**Fig 2:**
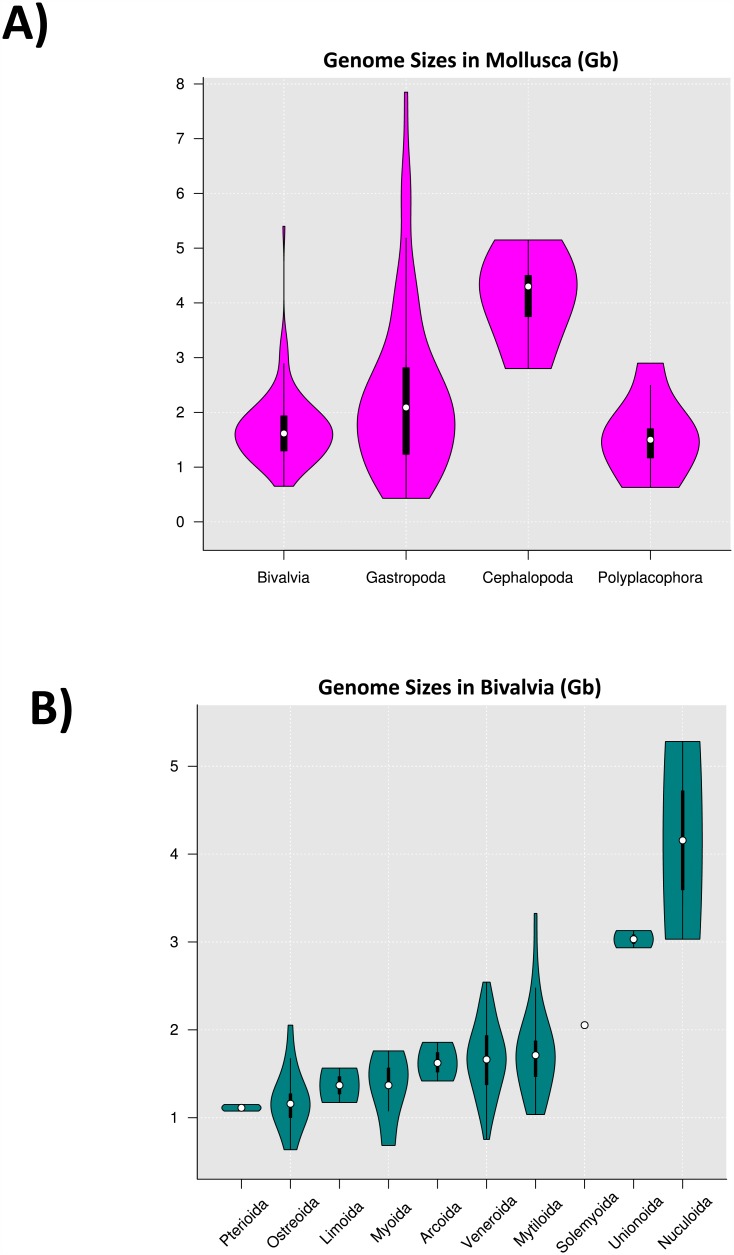
Genome size distribution of Molluscs (A) and Bivalves (B). Violin plots were built using DNA mass content data from Genome Size Animal Database (http://www.genomesize.com/) converted into number of base pairs. Kernel density for Solemyoida is absent since there is only one record in this group. Data for Pterioida comes from the genome size estimation of *P*. *fucata* and *Atrina rigida* (belonging to Mytiloida on the original dataset)

Conveniently, genome size can also be estimated from the k-mer count data [46]. Using this method, we estimated the genome size of *M*. *galloprovincialis* to be 1.6 Gb. Interestingly, discrepancies between genome sizes estimated from sequencing and experimental data have been previously reported [53]. Using flow cytometry, *M*. *galloprovincialis* (2n = 28 [54]) was proposed to have a genome size of either 1.4 Gb [55] or 1.9 Gb [56], while our 1.6 Gb estimate fits nicely in the middle. When compared with other bivalves, a 1.6 Gb genome size for *M*. *galloprovincialis* is located approximately in the middle tier not only among bivalves (Fig 2A) but also among other Mytiloida (Fig 2B). Moreover, the genome size range of Mytiloida is one of the highest within the superclass Pteriomorpha when sorted by their median. These relative positions of the *M*. *galloprovincialis*’ genome size highlights the representativeness of this genome size within the taxa included in our comparisons and the possibility to use this genome as a “model” for Mytiloida and Bivalvia taxa for some genomic features such as repeat elements and gene content.

### Assembly fragmentation in the *M*. *galloprovincialis* genome

In a genome sequencing survey, most of the biological information is obtained by analysing the final assembly. Base calls and quality values from reads of sequencing libraries with different insert sizes provide the information needed to assemble small reads into larger structures such as contigs and scaffolds. For *M*. *galloprovincialis*, we used the *de novo* assembler SOAPdenovo for genome assembly. This assembler has been previously used for *de novo* assembling dozens of plant and animal genomes, including panda[27], duck [57], potato [45] and cucumber [58], among others. In Table 1, we show assembly statistics of *M*. *galloprovincialis* and the other four marine molluscs. These statistics revealed a highly fragmented assembly. For instance, the assembly contained a large number of sequences (1,746,447) and low N50 (2,651 bp) when compared to *A*. *californica*, *P*. *fucata*, *C*. *gigas* and *L*. *gigantea* assemblies.

**Table 1:** Assembly statistics of *M*. *galloprovincialis* and four other molluscan genome sequencing projects. Data shown was obtained with the Assemblathon 2 metrics script.

	*M*. *galloprovincialis*	*C*. *gigas*	*P*. *fucata*	*L*. *gigantea*	*A*. *californica*
**Estimated Genome Size**	**1,600 Mb**	**545 Mb**	**1150 Mb**	**359,5 Mb**	**1,800 Mb**
**Number of scaffolds**	1,746,447	11,969	800,982	4,475	8,766
**Total size of scaffolds**	1,599,211,957	558,601,156	1,413,178,538	359,512,207	715,791,924
**Total scaffold length as percentage of known genome size**	100.0%	102.5%	122.9%	100.0%	39.8%
**Longest scaffold**	67,529	1,964,558	698,791	9,386,848	1,784,514
**Shortest scaffold**	100	100	100	1000	5,001
**Number of scaffolds > 500 nt**	676,492 (38.7%)	6,484 (54.2%)	323,197(40.4%)	4,475 (100.0%)	8,766 (100.0%)
**Number of scaffolds > 1K nt**	393,685 (22.5%)	5,788 (48.4%)	142,882 (17.8%)	4,471 (99.9%)	8,766 (100.0%)
**Number of scaffolds > 10K nt**	12,859 (0.7%)	3,172 (26.5%)	27,367 (3.4%)	1,318 (29.5%)	5,269 (60.1%)
**Number of scaffolds > 100K nt**	0 (0.0%)	1,353 (11.3%)	629 (0.1%)	291 (6.5%)	2,079 (23.7%)
**Number of scaffolds > 1M nt**	0 (0.0%)	60 (0.5%)	0 (0.0%)	98 (2.2%)	27 (0.3%)
**Mean scaffold size**	916	46,671	1,764	80,338	81,655
**Median scaffold size**	258	824	402	3,622	13,763
**N50 scaffold length**	2,651	401,319	14,455	1,870,055	264,327
**Percentage of assembly in scaffolded contigs**	18.5%	95.7%	75.4%	99.0%	93.9%
**Percentage of assembly in unscaffolded contigs**	81.5%	4.3%	24.6%	1.0%	6.1%
**Average number of contigs per scaffold**	1.1	2.8	1.3	4.1	7.4
**Sequencing Coverage**	32X	155X	40X	8,87X	11X
**Sequencing Technology**	Illumina	Illumina	454 + Illumina	Sanger	Sanger

Two reasons could explain the differences among assembly statistics for these genomes. The first one is genome size. The difficulty of assembling a genome increases with its size. Genomes of large sizes contain correspondingly a high number of internal repetitions such as paralogues, duplications, structural rearrangements, and mobile elements [59]. Reads from these DNA regions can match more than one genome position, decreasing the contiguity of assemblies. The genome size of *M*. *galloprovincialis* is only comparable with that of *A*. *californica*, while those of *P*. *fucata*, *C*. *gigas* and *L*. *gigantea* are 33, 66 and 75% smaller, respectively. The second reason is the sequencing technology used. For instance, the assemblies of *L*. *gigantea* and *A*. *californica*, despite their low coverage (8X and 11X, respectively), showed much better assembly statistics than that of *M*. *galloprovincialis*. Larger reads obtained by Sanger sequencing technology for these two genomes surely contributed to the lower number of scaffolds and larger N50 obtained. For *P*. *fucata*, with a final 35X coverage, both Illumina and 454 sequencing were used. However, despite using the same short-read sequencing technology as in *M*. *galloprovincialis*, the assembly statistics for *C*. *gigas* were superior. The assembly of this organism, using reads from Illumina mate-pair and pair-end libraries, was improved with the addition of fosmid libraries in the scaffolding step. In our case, using only pair-end sequencing libraries did prevent contigs from assembling into larger scaffold sequences.

### *Ab initio* prediction of repetitive sequences identifies a large diversity of repetitive elements

Repetitive elements (REs) are an important part of most eukaryotic genomes [60]. From humans to plants [61], a high proportion of these genomes consists of REs (i.e. interspersed repeats and low complexity DNA sequences). Although originally considered as "junk" DNA, they can play an important role in the adaptation [62] and evolution [63,64] of eukaryotes. To measure the extent of REs in the genome of *M*. *galloprovincialis*, we used RepeatMasker (Table 2) with Repbase and in-house repeat libraries. Using only Repbase, results of both “MYTILUS” and “MOLLUSCA” analyses found low repetitive content (1.4% and 1.57% respectively). However, identification of REs in a genome is not a trivial task. Difficulties arise when they do not share any similarity at sequence level with any other repeat sequences in curated databases as Repbase. To overcome this issue, *ab initio* methods (e.g. RepeatModeler) build libraries of new repeats [65] from scratch. The resulting libraries are then used to identify more precisely the repeat content of a genome. For example, using an *ab initio* prediction method, 36% of the *C*. *gigas* genome contained REs [24]. To test whether this high percentage of REs is a unique feature of *C*. *gigas*, we used RepeatModeler to build libraries of repetitive sequences in *M*. *galloprovincialis*. The INHOUSE search found 36.13% of REs in our assembly, corresponding to 1276 different families. Noteworthy, 30.16% of the genome corresponded to "Unclassified" *de novo* REs belonging to 1059 clusters. On the other hand, from the "classified" part, the most representative fraction (2.27% of the genome) was made of repetitive DNA elements.

**Table 2:** Percentage of bases masked in the assembly using different RepeatMasker libraries.

	% Bases masked			Families found (using RepeatModeler Library)
	RepeatMasker Own Species Library	RepeatMasker Mollusca Library	RepeatModeler Library	
***M*. *galloprovincialis***	1.40%	1.57%	36.13%	1276
***C*. *gigas***	2.71%	2.81%	31.90%	870
***P*. *fucata***	1.19%	1.37%	37.46%	1524
***L*. *gigantea***	1.37%	1.42%	22.47%	621
***A*. *californica***	11.45%	11.53%	43.70%	938

**Fig 3:**
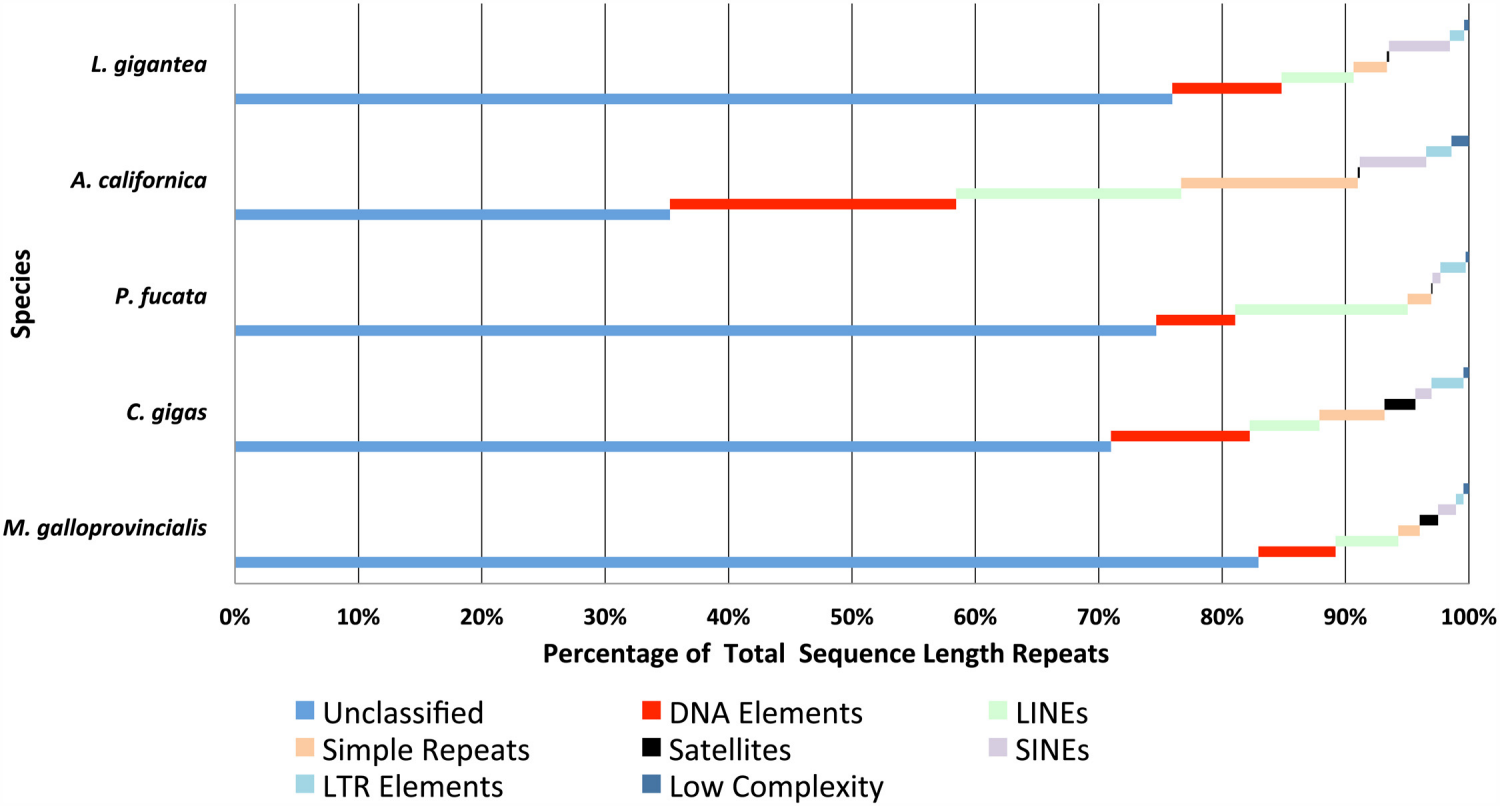
Percentage of each repetitive element relative to the total sequence length occupied by them.

Similar repeat content screenings were done in *C*. *gigas*, *L*. *gigantea*, *P*. *fucata* and *A*. *californica*. In these analyses, the organisms with more REs corresponded to *A*. *californica* with 43,70%, and the lowest to *L*. *gigantea* with 22.47%. Coincidentally, these organisms had the largest and smallest genome sizes, respectively. These results went in accordance with the long known positive correlation between genome size and REs content in eukaryotic genomes [66]. In *P*. *fucata*, this *ab initio* prediction of REs found almost four times (37.46%) as much as the percentage predicted using only the Repbase database [25]. Comparatively, the *M*. *galloprovincialis* genome contained proportionally the largest content (>80%) of unknown REs in Molluscs (Fig 3). Moreover, the genomes of *P*. *fucata* and *M*. *galloprovincialis* harboured the largest number of unknown families of REs among the molluscs studied: 1325 and 1059 respectively. One possible explanation for these high numbers would be that these unknown families are artefacts resulting from low coverage sequencing. However, a second possibility is that the unknown families come from multiple novel species-specific REs. Long-read sequencing over repeat-containing genomic regions will help to distinguish between these two alternative explanations.

A large group of REs in a genome are transposable elements (TEs). In our analyses, the molluscan genome with the largest content of DNA TEs and retrotransposons corresponded to *A*. *californica*, encompassing 10.15% and 11.23% of its genome, respectively. In *M*. *galloprovincialis*, the large proportion of unknown repetitive sequences made it difficult to extract conclusions about the diversity of TEs. On the other hand, the most abundant TEs belonged to the *Penelope* family [67] with 86,269 copies. The *Penelope* retrotransposon induces a hybrid dysgenesis syndrome in *Drosophila virilis*, maybe as a first step for reproductive isolation among populations [68]. In addition, we found only one transposon group significatively more abundant in *M*. *galloprovincialis* than in the other molluscs studied, the DNA transposon Tip100 family [69]. This transposon family belongs to the hobo-*Ac*-Tam3 (hAT) superfamily. Interestingly, hobo transposons are also involved in hybrid dysgenesis in *D*. *melanogaster* [70]. Elliot and Gregory [71] proposed that larger genomes evolve primarily through the expansion of only a small subset of existing TEs. The Tip100 family can be part of this subset of TEs in our genome. Further studies are needed to test whether members of *Penelope* or Tip100 families work similarly in *M*. *galloprovincialis*.

### Mitochondrial genomes

Mitochondria are essential components of the cell where they produce energy through oxidative phosphorylation. In addition, they can also mediate phenotypes such as lifespan, fertility, starvation resistance, altitude adaptation, and temperature regulation [72]. Conveniently, genome sequencing data in eukaryotes contain also mitochondrial genome sequences. This is because methods for DNA/RNA isolation capture also organelle nucleic acids. For instance, a bioinformatic pipeline has been recently proposed to extract mitochondrial reads from genome sequencing data and assemble them in organisms without a reference mitochondrial genome [73]. Two types of mitochondrial genomes, female (MgF) and male (MgM), have been reported for *M*. *galloprovincialis* [74]. BLASTN and *nucmer* alignments of the assembly against MgF produced significant matches with 56 (67.6% of coverage) and 51 (68.5% of coverage, S1 Fig) contigs, respectively. In addition BLASTN and *nucmer* alignments of the assembly against MgM produced a low number of significant matches with only 9 (22,2% of coverage) and 9 (20.5% of coverage) contigs, respectively. The incomplete coverage of both MgF and MgM could be explained by the high stringent conditions used in the assembly (due to the heterozygosity of the *M*. *galloprovincialis* genome). These conditions may have prevented the assembler to behave normally in the presence of reads from two rather similar mitochondrial genomes and in different proportions within the studied tissue. Indeed, this incomplete coverage may be also due to the low similarity between our mitochondrial sequences in the assembly with MgF and MgM.

**Fig 4:**
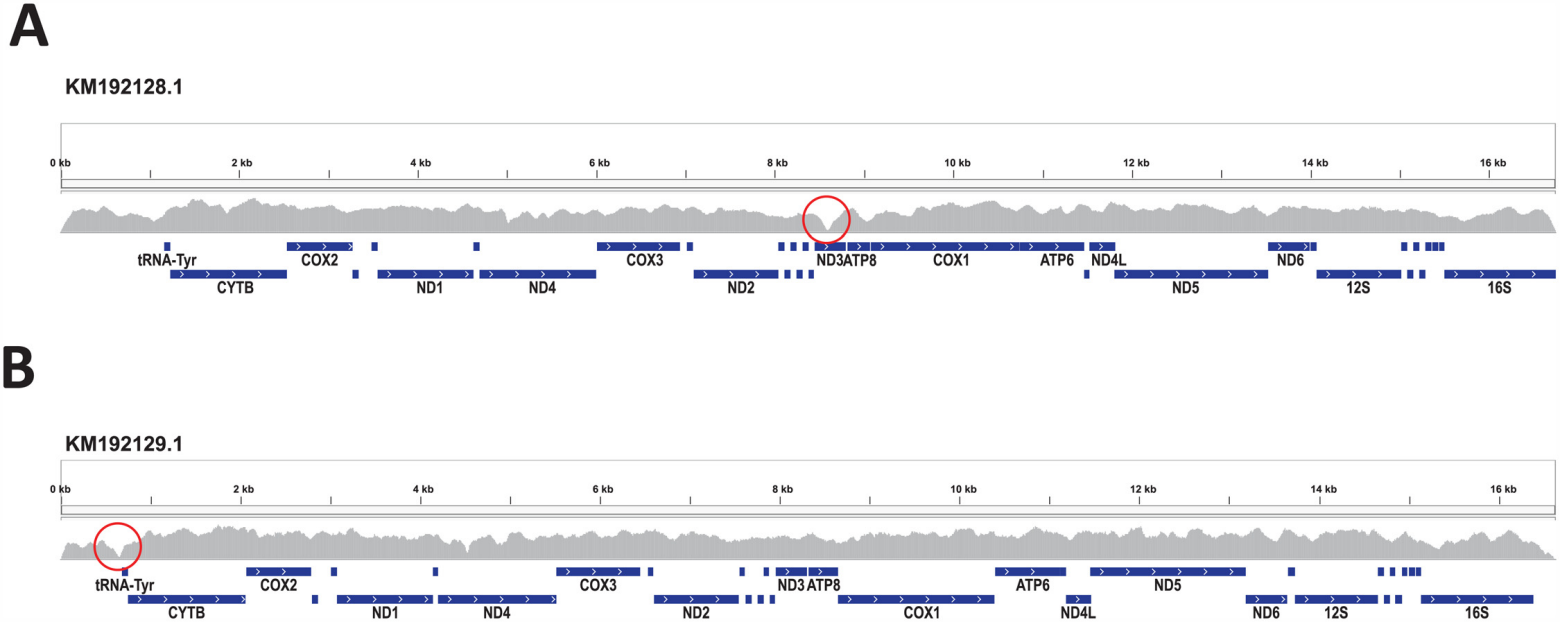
Read mapping against the mitochondrial genomes A) MeF (GenBank KM192128) and B) MeM (Genbank KM192129). Red circles highlight the regions of least coverage.

To test the latter possibility, we searched further for mitochondrial sequences using the repertoire of reads from *M*. *galloprovincialis*. We directly mapped them onto different mitochondrial genomes from the genus *Mytilus*. Read mapping against mitochondrial genomes of *Mytilus* spp. produced a full coverage of several mitochondrial genomes. The largest number of reads (113,824) mapped onto an F mitochondrial genome from *M*. *edulis* (MeF, GenBank KM192128, Fig 4A), slightly more (111 more) than the number of reads that mapped to MgF. Moreover, fewer variants were found when mapping against MeF (228 variants) than MgF (302 variants). These two mitochondrial genomes were 99,26% identical to each other at nucleotide level. Surprisingly, a well-covered mapping against an M mitochondrial genome of *M*. *edulis* (MeM, Genbank KM192129) was also observed (Fig 4B). The average mapping coverage against the latter was 195X, about 10 times lower than that against MeF (1156X). A likely explanation of a better read mapping over *M*. *edulis* mitochondrial genomes is introgression. Introgressed *M*. *edulis* mitochondrial haplotypes in *M*. *galloprovincialis* and *M*. *trossulus* populations have already been described [75,76]. Śmietanka et al. [76] reported a predominance of *M*. *edulis* mitochondrial haplotypes in the Atlantic *M*. *galloprovincialis* population. On the other hand, despite the presence of both M and F haplotypes in our data, it was not possible to determine the sex of our sequenced individual based only on its mitochondrial sequences. Though individuals having both M and F haplotypes are most likely males [3,77], presence of the M haplotype is not causally linked to masculinity [78]. Moreover, presence of M haplotypes in female individuals outside of hybrid zones due to disruption of DUI has also been reported [79].

Read mapping was not evenly distributed along mitochondrial genomes. Sequence motifs such as inverted repeats and GGC motifs could produce some coverage bias [80]. When mapping our reads against MeM, the region least covered (18X) corresponded to a short sequence in the control region (CR), upstream of the tRNA^Tyr^ gene, within its corresponding variable domain 2 (VD2) [81]. This could be due to the presence of homopolymers of guanines in VD2 that could have biased the sequencing step [80]. On the other hand, the region least covered of MeF was located within a coding gene, the NADH dehydrogenase 3 (ND3) (Fig 4A). However, this region did not contain homopolymers. One explanation for this low coverage could be the presence of DNA secondary structures in this region. Illumina technology is vulnerable to bias due to secondary structures formed during the amplification step [82]. Rodakis et al. [83] postulated the presence of a hairpin at positions 8545 to 8573 of MgF, only 9 bp from the start of the corresponding region of low coverage. This hairpin structure might be the origin of replication of the light strand in F haplotypes under the asymmetrical model of mtDNA replication. Though this hairpin structure is not located exactly over the low covered region, its closeness may have biased the amplification and sequencing of the DNA fragment covering this mitochondrial region (Fig 4A).

Although mitochondrial evolution is usually assumed to be neutral, evidence of selective pressures on mitochondria has been reported in different organisms [84–87]. Relevance of mitochondrial nucleotide variation on the fitness of individuals has been a matter of dispute. To characterize this variation, we searched for single-nucleotide variants (SNVs) within our mitochondrial DNA sequences. Variant calling using MeF showed that 192 out of 228 (84,95%) variants were located in coding regions. Both M and F mitochondria in *M*. *galloprovincialis* contain two rRNA, 13 protein-coding, and 23 tRNA genes. In our mapping over MeF, the gene containing the highest ratio of variants was ND4 (S1 Table). Interestingly, ND4 was previously reported as the most abundant expressed transcript in mussels [88]. Thus, the high ratio of variants in ND4 agreed with previous evidence of elevated number of mutation rates in highly expressed genes [89,90]. However, adaptive processes could produce some of these ND4 variants. Signatures of adaptive variation on this proton pump were reported in other marine organisms [84,91]. On the other hand, when mapping our reads against MeM, the coding gene with the highest percentage of variants per nucleotide was cytochrome b (CYTB) gene. This gene has been previously reported to be downregulated on male mussels when exposed to 17beta-estradiol E2 [92]. In mussels, the adaptive variants in ND4 and CYTB could regulate the pH gradient affecting respiratory control. Further studies need to be done to test the role of these genes in the hypoxia tolerance of mussels in the intertidal zone.

### Gene models

One of the major objectives of a genome-sequencing project is gene prediction. Due to the low sequencing depth of *M*. *galloprovincialis* (32X), we expected that the corresponding assembly would be either incomplete or extensively fragmented. Accordingly, gene prediction in our assembled genome sequencing data yielded an incomplete gene repertoire. To assess the amount of missing genes in our working assembly, the completeness of the gene content was measured with CEGMA [38]. Results of the CEGMA analyses showed that only 39 (15.73%) of the 248 core eukaryotic genes (CEGs) were considered “complete” in our assembly. When the CEGMA analysis was extended to include also partial but significant matches, 107 (43.27%) proteins aligned. This percentage is approximately three times as much as the number of complete CEGs found. Table 3 shows the result of the completeness analyses in other mollusc genomes using the same approach. *L*. *gigantea* showed the highest percentage of completeness (85.89%). This may be explained by its reduced genome size and the use of Sanger technology for sequencing. Second was *C*. *gigas*, where about three quarters of its genes (78.63%) gave “complete” matches. Noteworthy, in *M*. *galloprovincialis* and *P*. *fucata* the percentage of "partial" matches were approximately three times as much as the number of "complete" CEGs. This observation could be explained also by the low coverage sequencing of these two genomes.

**Table 3:** Results for the CEGMA completeness analyses for 5 molluscan species.

Species	Completeness			Genome Size	Genome Coverage
		Proteins	Percentage		
***M*. *galloprovincialis***	**Complete**	39	15.73%	1,600 Mb	40X
	**Partial**	107	43.27%		
***C*. *gigas***	**Complete**	195	78.63%	545 Mb	155X
	**Partial**	236	95.16%		
***P*. *fucata***	**Complete**	63	25.00%	1,150 Mb	40X
	**Partial**	153	61.69%		
***L*. *gigantean***	**Complete**	213	85.89%	359,5 Mb	8.87X
	**Partial**	242	97.58%		
***A*. *californica***	**Complete**	90	36.29%	1,800 Mb	11X
	**Partial**	207	83.47%		

Fortunately, a preliminary view of the gene repertoire in *M*. *galloprovincialis* may be informative enough even at low sequencing depth. This repertoire consisted of 10,891 protein-coding genes. In addition, we can extrapolate the percentage of “partial” mapped CEGs as the completeness of the whole gene repertoire in our genome assembly [38]. Therefore, the expected number of genes in the *M*. *galloprovincialis* genome would be about 25,000 genes.

**Fig 5:**
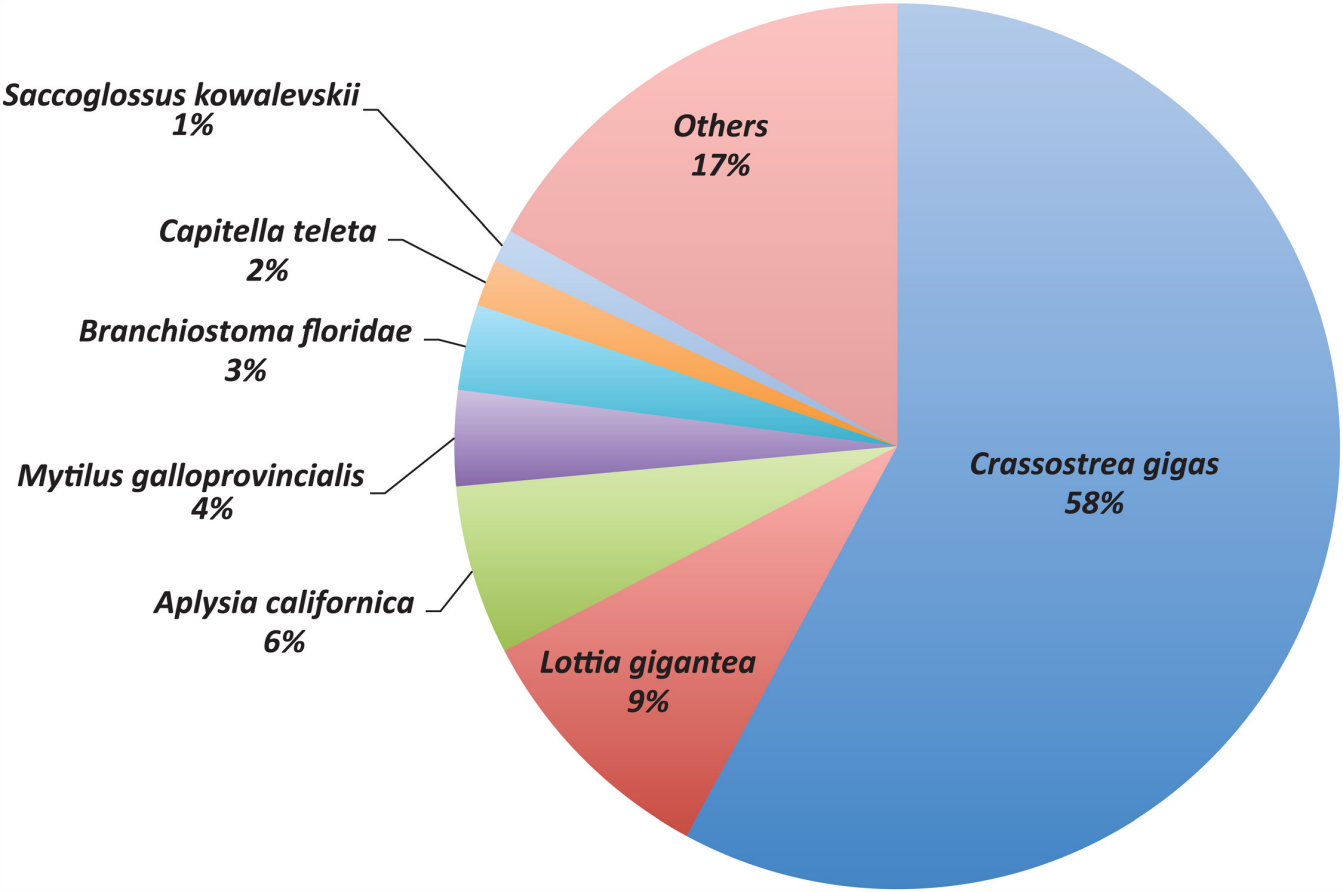
Distribution of best protein BLAST hits by species. Only species appearing in more than 1% of BLAST hits are represented.

Finally, although the Blast2GO annotation resulted in 2,397 predicted protein sequences with no BLAST hits against the non-redundant protein database, it succeeded in effectively annotating about a quarter (2,800 sequences) of the total number of proteins in our assembly. The species that contributed most annotations was *C*. *gigas* (Fig 5).

### Gene Functional Annotation

**Fig 6:**
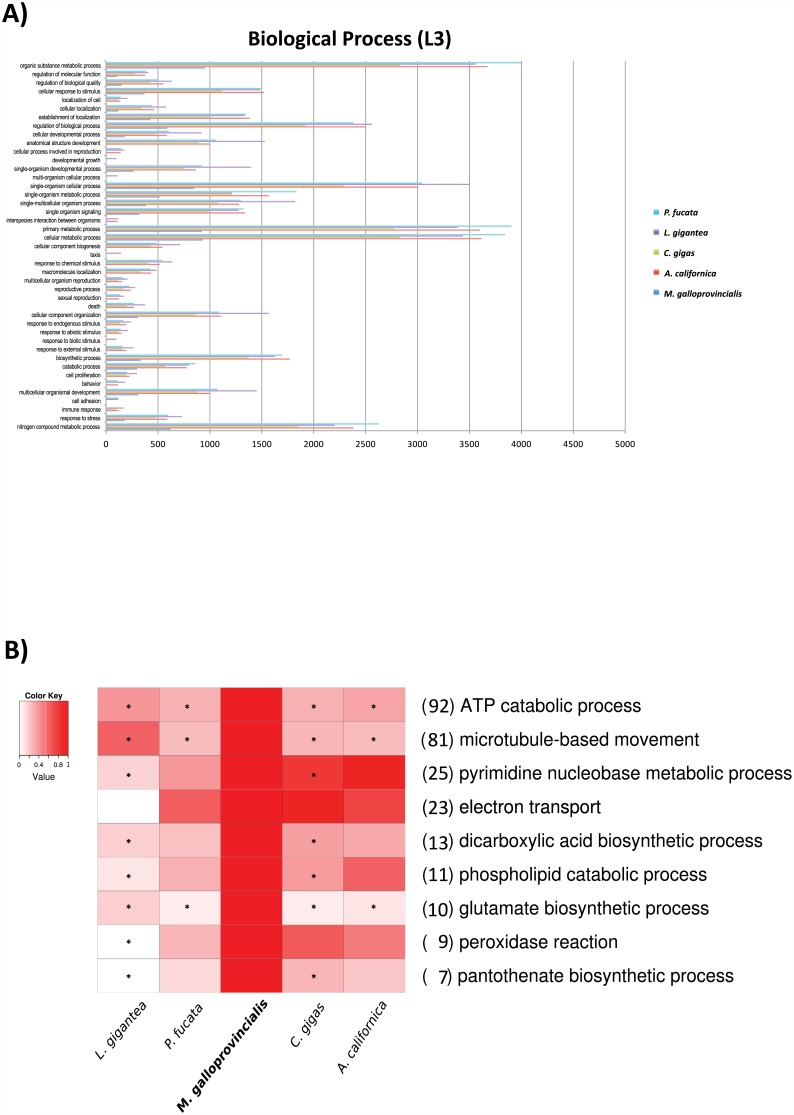
**A)** Gene Ontology predictions involved in biological processes at Level Three for the mollusc genomes studied. **B)** Biological Process Gene Ontologies with significant differences between *M*. *galloprovincialis* and other molluscs. Heatmap shows those ontologies where *M*. *galloprovincialis* contained the largest percentage of genes per genome. Heatmap values were normalized assigning value one to *M*. *galloprovincialis*. Numbers next to each GO term indicate the number of genes in mussels with that annotation. Asterisks indicate the genome comparisons where mussel genes are statistically overrepresented as obtained with Blast2GO.

In the ontology assignments of Blast2GO there was not any biological process category at level three overrepresented in *M*. *galloprovincialis* relative to the other molluscs (Fig 6A). The most abundant level-three gene ontology (GO) terms found in the other molluscs corresponded also to the most abundant GO terms found in *M*. *galloprovincialis*: organic substance metabolic process (GO:0071704, 953 genes), cellular metabolic process (GO:0044237, 931 genes) and single-organism cellular process (GO:0044763, 924 genes). However, half of the common level-three GO terms (21 out of 43 genes) in other molluscs were not found in *M*. *galloprovincialis*. The most likely explanation was the limited completeness of our genome.

Despite the completeness limitation, we were able to find genes with functional annotations that would explain how mussels cope with the specific characteristics of their environment, such as those related to immunity, resistance to hypoxia, shell formation, and adhesion to surfaces (S2 File). First, we looked for genes with immune-related functions in mussels that provide defence against bacteria and other environmental challenges [93–95]. GO terms related to these functions were “immune response” (GO:0006955, twenty genes), “immune system process”(GO:0002376, fifty five genes), and “defence response” (GO:0006952, thirty five genes). These genes had functions related to toll-like receptor signalling pathway, inflammatory response, regulation of innate immune response, and defence response to fungus, virus and bacteria. Second, other genes important for adaptation in mussels are genes that confer resistance to hypoxia and oxidative stress during tidal emersion and resubmersion cycles [96,97]. We found seven genes annotated with the GO term “resistance to hypoxia” (GO:0001666), such as isocitrate dehydrogenase [98] and ubiquitin carboxyl-terminal hydrolase [99] genes. In addition, another 25 genes were annotated with the GO term “response to oxidative stress” (GO:0006979). Finally, we manually searched for genes that provide physical protection against the environment. We found genes involved in byssus attachment to surfaces [100] and shell formation [101] (S2 File). In summary, our *M*. *galloprovincialis* genome contained genes whose functions may help this organism to adapt to its environment. They provide a starting point to test experimentally their role in these functions and their relevance in mussel biology.

Contrasting the GO annotations of *M*. *galloprovincialis* with those of other molluscs can reveal unique characteristics of the former. One hundred and forty ontologies presented significant differences in number (either positive or negative) in the *M*. *galloprovincialis*-versus-all comparison (S2 Table). In three of these ontologies mussel genes were significatively overrepresented (Fig 6B). The first of these three corresponded to GO:0006200, which is related to energy production based on ATP consumption. There were two main groups of gene functions within this GO term in *M*. *galloprovincialis*: ATP-binding cassette (ABC) (eight genes) and multidrug resistance-associated (16 genes). Interestingly, ABC transporters in *Mytilus* spp. [102] and other organisms [103–105] were previously reported as protection against multixenobiotics. These genes would represent the first line of defence against natural and anthropogenic toxicants in the marine environment. The second overrepresented GO term corresponded to “glutamate biosynthetic process” (GO:0006537, ten genes). Five of these were glutamate synthase genes. Similar annotations were also found in transcripts from hepatopancreas in crustaceans [106]. Glutamate synthase is mainly involved in the synthesis of glutamic acid from its precursor α-ketoglutarate [107]. Finally, the third overrepresented GO term corresponded to “microtubule-based movement” (GO:0007018). Almost half of the 81 proteins in this GO category were annotated as axonemal dynein heavy chain. The second most abundant gene function within this GO term corresponded to kinesin, a motor protein [108]. Both axonemal dyneins and kinesins participate in the active transport of molecules along ciliary structures [109,110].

At least two of the three aforementioned overrepresented ontologies in *M*. *galloprovincialis* had annotations linked to specific biological functions relevant for *M*. *galloprovincialis*. Indeed, genes contributing to either maintenance of ciliary structures (GO:0007018) or multixenobiotic resistance (GO:0006200) [111,112] might be subject to high selective pressures from the environment. Therefore, the multiplicity of these genes in its gene repertoire might represent a genomic adaptation of *M*. *galloprovincialis* to sedentary filter-feeding life style that forces them to deal with a variety of changing environments and ecological characteristics. Further studies of the gene content, annotation and expression of these genes in *M*. *galloprovincialis* should be carried out to validate this hypothesis.

## Conclusion

Next-Generation Sequencing (NGS) technologies have already significantly increased our understanding of many genomes across the Tree of Life. Next-Generation Sequencing has the potential to increase our basic knowledge on the genomes of non–model organisms such as marine molluscs, where genomic resources are scarce. As shown here with the Mediterranean mussel *Mytilus galloprovincialis*, *de novo* genome surveys at low-level sequencing depth can be used to provide first insights into the composition and structure of genomes in non-model organisms [18,19]. This study has shed some light onto the genome complexity, abundance of REs and (partial) gene repertoire of *M*. *galloprovincialis*. The comparative analyses of the genomic features observed in *M*. *galloprovincialis* with other marine molluscs have shown that an important part of the genome in these organisms contains a large number of repetitive sequences. Most of the REs found in *M*. *galloprovincialis* are unknown and need to be quantified and classified in more detail. Moreover, our analysis of the gene content in *M*. *galloprovincialis* has put into evidence the limits of low sequencing depth projects for gene annotation in complex genomes. Despite these limitations, through comparison with other molluscan genomes, we managed to identify two biological functions, detoxification and ciliary structure maintenance, where *M*. *galloprovincialis* has a large number of genes, most likely as a consequence of its condition of filter-feeder. This low-coverage genome survey will help in the design of additional sequencing and novel assembly strategies to obtain a more complete view of the mussel genome and the evolutionary forces that may have shaped its architecture and composition.

## Availability of Supporting Data

Illumina read sequences used in this study can be downloaded from the NCBI Sequence Read Archive under the accessions SRR1598987, SRR1598945 and SRR1598943. Assembled sequences and annotations have been submitted to NCBI under the submission code SUB1006464 (PRJNA262617) following NCBI WGS requirements.

## Supporting Information

S1 Fig Nucmer alignments over the Mitochondrial F haplotype of *M*. *galloprovincialis*. Rectangles depict direct (red) and reverse (blue) matches.
(PDF)

S1 File Assemblies and Mitogenomes–List of web sites containing genome assemblies of the studied molluscs and GenBank accession numbers of all the mitochondrial genome sequences analysed. (PDF)

S2 File Summary of gene functional annotations. List of genes with functions related to immunity, resistance to hypoxia and stress, shell formation and adhesion to surfaces.
(PDF)

S1 Table Variants in mitochondrial genes. The table shows the number of variants present in the mitochondrial genes. The following information is given: gene name, size of the gene in base-pairs, absolute number of variants found and percentage of variants related to the gene length.
(PDF)

S2 Table GO terms under the category “biological process” with significant differences between *M*. *galloprovincialis* and at least one mollusc protein set. The following information is given: GO-ID, GO-term, Number of proteins in the organism for that GO annotation, False Discovery Rate value, P-value and whether it is up or downregulated. For those non-significant terms, the symbol ⌘ is used. When significant, “++” is used for proteins overrepresented in *M*. *galloprovincialis*, and “–-” for those underrepresented.
(PDF)
