# Correction: A First Insight into the Genome of the Filter-Feeder Mussel *Mytilus galloprovincialis*

**DOI:** 10.1371/journal.pone.0160081

**Published:** 2016-07-21

**Authors:** Maria Murgarella, Daniela Puiu, Beatriz Novoa, Antonio Figueras, David Posada, Carlos Canchaya

A reference is omitted from the fourth and seventh sentences of the second paragraph under the sbuheading “Mitochondrial genomes” in the Results and Discussion section.

The fourth sentence should read: The largest number of reads (113,824) mapped onto an F mitochondrial genome from *M*. *edulis* (MeF, GenBank KM192128, Zbawicka et al., 2014, Fig 4A), slightly more (111 more) than the number of reads that mapped to MgF.

The seventh sentence should read: Surprisingly, a well-covered mapping against an M mitochondrial genome of *M*. *edulis* (MeM, Genbank KM192129, Zbawicka et al., 2014) was also observed (Fig 4B).

The reference is: Zbawicka M, Wenne R, Burzyński A. Mitogenomics of recombinant mitochondrial genomes of Baltic Sea *Mytilus* mussels. Mol Genet Genomics. 2014;289: 1275–1287. doi:10.1007/s00438-014-0888-3.
