# Supplementary figures and images for: A First Insight into the Genome of the Filter-Feeder Mussel Mytilus galloprovincialis
Source: PLoS One. 2016 Mar 15;11(3):e0151561. doi: 10.1371/journal.pone.0151561 (PMC4792442; doi:10.1371/journal.pone.0151561)

# S1 Figure

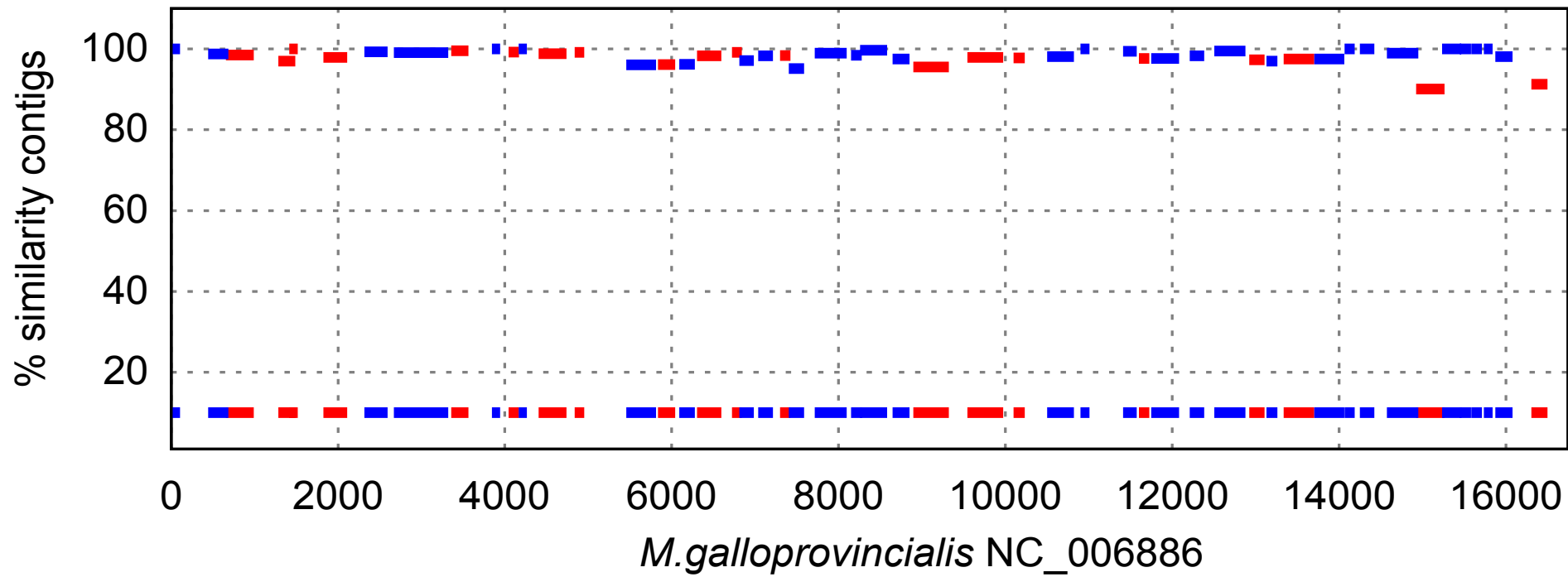

Supplement: S1 Fig — Rectangles depict direct (red) and reverse (blue) matches. (PDF) [file pone.0151561.s001.pdf]
