# Supplementary material for: A First Insight into the Genome of the Filter-Feeder Mussel Mytilus galloprovincialis
Source: PLoS One. 2016 Mar 15;11(3):e0151561. doi: 10.1371/journal.pone.0151561 (PMC4792442; doi:10.1371/journal.pone.0151561)
Supplement: S1 File — (PDF) [file pone.0151561.s002.pdf]

S1 File

List of web sites with genome assembly data of the four other studied molluscs

1. *Crassostrea gigas* (“Pacific oyster”)
   http://gigadb.org/dataset/100030
2. *Pinctada fucata* (“pearl oyster”)
   http://marinegenomics.oist.jp/pearl/viewer/download?project_id=20
3. *Lottia gigantea* (“owl limpet”)
   http://genome.jgi.doe.gov/Lotgi1/Lotgi1.download.html
4. *Aplysia californica* (“California sea hare”)
   http://www.broadinstitute.org/ftp/pub/assemblies/invertebrates/aplysia/AplCal2/

List of GenBank accession numbers of all the mitochondrial genome sequences analysed.

1. AY363687
2. AY484747
3. AY497292
4. AY823623
5. AY823624
6. AY823625
7. DQ198225
8. DQ198231
9. DQ399833
10. EF434638
11. EF434638
12. FJ890849
13. FJ890850
14. GQ438250
15. GQ527172
16. GQ527173
17. GU936625
18. GU936626
19. GU936627
20. HM462080
21. HM462081
22. JX486123
23. JX486124
24. KJ577549
25. KM192124
26. KM192125
27. KM192126
28. KM192127
29. KM192128
30. KM192128
31. KM192128
32. KM192129
33. KM192130
34. KM192131
35. KM192132
36. KM192133
37. KM192134
38. NC_006161
39. NC_006886
40. NC_006886
41. NC_006886
42. NC_007687
43. NC_015993
44. NC_024733
