# Supplementary material for: A First Insight into the Genome of the Filter-Feeder Mussel Mytilus galloprovincialis
Source: PLoS One. 2016 Mar 15;11(3):e0151561. doi: 10.1371/journal.pone.0151561 (PMC4792442; doi:10.1371/journal.pone.0151561)
Supplement: S2 File — List of genes with functions related to immunity, resistance to hypoxia and stress, shell formation and adhesion to surfaces. (PDF) [file pone.0151561.s003.pdf]

S2 File - Summary of gene functional annotations

Table 1 – Antimicrobial Peptide genes

Name	Number of proteins	Protein name
**Myticin**	3	Mga_v1-02092;Mga_v1-04730;Mga_v1-05471
**Mytimycin**	1	Mga_v1-09938
**Hydramacin**	2	Mga_v1-03014;Mga_v1-06767

Table 2 - Immunity-related GO terms used for search

GO term	GO ID	Number of proteins	Protein name
**Immune Response**	**GO: 0006955**	20	Mga_v1-00408;Mga_v1-00487;Mga_v1-00790;Mga_v1-01218;Mga_v1-02879;Mga_v1-03963;Mga_v1-03965;Mga_v1-05364;Mga_v1-05586;Mga_v1-06041;Mga_v1-06899;Mga_v1-06935;Mga_v1-07007;Mga_v1-07614;Mga_v1-07995;Mga_v1-08031;Mga_v1-08210;Mga_v1-08708;Mga_v1-09300;Mga_v1-1069
**Immune System Process**	**GO: 0002376**	55	Mga_v1-00177;Mga_v1-00408;Mga_v1-00452;Mga_v1-00487;Mga_v1-00790;Mga_v1-00901;Mga_v1-00925;Mga_v1-00926;Mga_v1-00927;Mga_v1-01218;Mga_v1-01716;Mga_v1-01886;Mga_v1-02213;Mga_v1-02879;Mga_v1-03519;Mga_v1-03963;Mga_v1-03965;Mga_v1-04068;Mga_v1-04163;Mga_v1-04998;Mga_v1-05026;Mga_v1-05085;Mga_v1-05364;Mga_v1-05586;Mga_v1-05589;Mga_v1-05737;Mga_v1-06041;Mga_v1-06203;Mga_v1-06374;Mga_v1-06708;Mga_v1-06739;Mga_v1-06821;Mga_v1-06899;Mga_v1-06935;Mga_v1-07007;Mga_v1-

			07122;Mga_v1-07332;Mga_v1-07377;Mga_v1-07404;Mga_v1-07614;Mga_v1-07910;Mga_v1-07995;Mga_v1-08031;Mga_v1-08210;Mga_v1-08342;Mga_v1-08708;Mga_v1-08883;Mga_v1-09294;Mga_v1-09300;Mga_v1-09656;Mga_v1-10011;Mga_v1-10198;Mga_v1-10199;Mga_v1-10294;Mga_v1-10694
**Defence response**	**GO: 0006952**	35	Mga_v1-00235;Mga_v1-00324;Mga_v1-00408;Mga_v1-00487;Mga_v1-00513;Mga_v1-00790;Mga_v1-01886;Mga_v1-02092;Mga_v1-02879;Mga_v1-02890;Mga_v1-03014;Mga_v1-03963;Mga_v1-03965;Mga_v1-04068;Mga_v1-04269;Mga_v1-04444;Mga_v1-04730;Mga_v1-04998;Mga_v1-05238;Mga_v1-05471;Mga_v1-06041;Mga_v1-06767;Mga_v1-06801;Mga_v1-06821;Mga_v1-06899;Mga_v1-07007;Mga_v1-07377;Mga_v1-07614;Mga_v1-07995;Mga_v1-08031;Mga_v1-08708;Mga_v1-09300;Mga_v1-09379;Mga_v1-09656;Mga_v1-10694

Table 3 - Response to Hypoxia and Response to stress related GO terms used for search

GO term	GO ID	Number of proteins	Protein name
**Response to Hypoxia**	**GO:0001666**	7	Mga_v1-00511;Mga_v1-00925;Mga_v1-00926;Mga_v1-05181;Mga_v1-07377;Mga_v1-10294;Mga_v1-10366
**Response to Oxidative Stress**	**GO:0006979**	24	Mga_v1-00404;Mga_v1-00535;Mga_v1-01395;Mga_v1-02122;Mga_v1-02221;Mga_v1-02384;Mga_v1-02890;Mga_v1-02976;Mga_v1-03682;Mga_v1-03897;Mga_v1-05586;Mga_v1-06585;Mga_v1-06789;Mga_v1-06949;Mga_v1-07377;Mga_v1-07653;Mga_v1-07654;Mga_v1-07747;Mga_v1-08528;Mga_v1-08673;Mga_v1-08923;Mga_v1-09253;Mga_v1-09297;Mga_v1-09412
**Response to Abiotic Stimulus**	**GO:0009628**	55	Mga_v1-00117;Mga_v1-00171;Mga_v1-00210;Mga_v1-00511;Mga_v1-00535;Mga_v1-00566;Mga_v1-00925;Mga_v1-

			00926;Mga_v1-00927;Mga_v1-00979;Mga_v1-01034;Mga_v1-01395;Mga_v1-01886;Mga_v1-02241;Mga_v1-02384;Mga_v1-02778;Mga_v1-02890;Mga_v1-03497;Mga_v1-03973;Mga_v1-04032;Mga_v1-04534;Mga_v1-04624;Mga_v1-04626;Mga_v1-04656;Mga_v1-04918;Mga_v1-05181;Mga_v1-05586;Mga_v1-05606;Mga_v1-06098;Mga_v1-06151;Mga_v1-06203;Mga_v1-06278;Mga_v1-06708;Mga_v1-06720;Mga_v1-06739;Mga_v1-06789;Mga_v1-06990;Mga_v1-07116;Mga_v1-07377;Mga_v1-07448;Mga_v1-07747;Mga_v1-08384;Mga_v1-08571;Mga_v1-08947;Mga_v1-08978;Mga_v1-09134;Mga_v1-09297;Mga_v1-09300;Mga_v1-09550;Mga_v1-10052;Mga_v1-10078;Mga_v1-10292;Mga_v1-10294;Mga_v1-10366;Mga_v1-10624

Table 4 - Response to Hypoxia and Response to Stress related genes

Category	Number of proteins	Protein name
**Actin**	3	Mga_v1-01022;Mga_v1-04003;Mga_v1-04176
**Cytochrome P450**	11	Mga_v1-01008;Mga_v1-03818;Mga_v1-05118;Mga_v1-05796;Mga_v1-06186;Mga_v1-06600;Mga_v1-07081;Mga_v1-07119;Mga_v1-09406;Mga_v1-09843;Mga_v1-09865
**Glutathione S-transferase**	5	Mga_v1-05695;Mga_v1-06031;Mga_v1-06512;Mga_v1-07193;Mga_v1-08862
**Superoxide dismutase**	3	Mga_v1-02456;Mga_v1-10163;Mga_v1-10682
**Adenosine deaminase**	4	Mga_v1-03325;Mga_v1-04043;Mga_v1-04484;Mga_v1-07706

Table 5 - Adhesion-related genes

Category	Number of proteins	Protein name
**Collagen**	10	Mga_v1-00278;Mga_v1-00312;Mga_v1-00971;Mga_v1-01705;Mga_v1-02407;Mga_v1-04145;Mga_v1-07105;Mga_v1-07687;Mga_v1-08152;Mga_v1-10229

Table 6 - Shell formation related genes

Category	Number of proteins	Protein name
**Chitin**	17	Mga_v1-00307;Mga_v1-00438;Mga_v1-00736;Mga_v1-01916;Mga_v1-01919;Mga_v1-03244;Mga_v1-03388;Mga_v1-03943;Mga_v1-04030;Mga_v1-06178;Mga_v1-06384;Mga_v1-06769;Mga_v1-07609;Mga_v1-08913;Mga_v1-10250;Mga_v1-10553;Mga_v1-10865
**Fibronectin**	1	Mga_v1-10338
**Laminin**	3	Mga_v1-06729;Mga_v1-06804;Mga_v1-10012
**Perlucin**	2	Mga_v1-01612;Mga_v1-05146
**Perlwapin**	1	Mga_v1-07535

Table 7 – Overrepresented GO categories in Biological Process

Category	Number of Proteins	Protein Name
ATP Catabolic Process (GO:0006200)	92	Mga_v1-00517;Mga_v1-00535;Mga_v1-00679;Mga_v1;00680;Mga_v1-00684;Mga_v1-00731;Mga_v1-00771;Mga_v1-00914;Mga_v1-

		00984;Mga_v1-01164;Mga_v1-01290;Mga_v1-01318;Mga_v1-01395;Mga_v1-01434;Mga_v1-01561;Mga_v1-01798;Mga_v1-01803;Mga_v1-02004;Mga_v1-02027;Mga_v1-02325;Mga_v1-02487;Mga_v1-02591;Mga_v1-02652;Mga_v1-02690;Mga_v1-02719;Mga_v1-02974;Mga_v1-03124;Mga_v1-03580;Mga_v1-03635;Mga_v1-03707;Mga_v1-04053;Mga_v1-04222;Mga_v1-04408;Mga_v1-04409;Mga_v1-04410;Mga_v1-04466;Mga_v1-04509;Mga_v1-04530;Mga_v1-04616;Mga_v1-04622;Mga_v1-04737;Mga_v1-04807;Mga_v1-04837;Mga_v1-05028;Mga_v1-05102;Mga_v1-05132;Mga_v1-05208;Mga_v1-05221;Mga_v1-05263;Mga_v1-05328;Mga_v1-05494;Mga_v1-05578;Mga_v1-06090;Mga_v1-06210;Mga_v1-06264;Mga_v1-06351;Mga_v1-06443;Mga_v1-06782;Mga_v1-06799;Mga_v1-06948;Mga_v1-07071;Mga_v1-07411;Mga_v1-07487;Mga_v1-07514;Mga_v1-07552;Mga_v1-07569;Mga_v1-07638;Mga_v1-07959;Mga_v1-08247;Mga_v1-08336;Mga_v1-08554;Mga_v1-08616;Mga_v1-08773;Mga_v1-08852;Mga_v1-08878;Mga_v1-09086;Mga_v1-09092;Mga_v1-09093;Mga_v1-09214;Mga_v1-09329;Mga_v1-09612;Mga_v1-09613;Mga_v1-09775;Mga_v1-09839;Mga_v1-09904;Mga_v1-10118;Mga_v1-10129;Mga_v1-10365;Mga_v1-10436;Mga_v1-10564,Mga_v1-10629;Mga_v1-10650
Microtubule-based Movement (GO:0007018)	81	Mga_v1-00097;Mga_v1-00517;Mga_v1-00771;Mga_v1-00914;Mga_v1-01164;Mga_v1-

		01290;Mga_v1-01318;Mga_v1-01545;Mga_v1-01546;Mga_v1-01803;Mga_v1-02004;Mga_v1-02027;Mga_v1-02325;Mga_v1-02487;Mga_v1-02591;Mga_v1-02690;Mga_v1-02719;Mga_v1-03124;Mga_v1-03208;Mga_v1-03580;Mga_v1-03625;Mga_v1-03654;Mga_v1-03866;Mga_v1-04222;Mga_v1-04410;Mga_v1-04509;Mga_v1-04530;Mga_v1-04807;Mga_v1-04849;Mga_v1-05102;Mga_v1-05132;Mga_v1-05221;Mga_v1-05226;Mga_v1-05263;Mga_v1-05328;Mga_v1-05383;Mga_v1-05450;Mga_v1-05494;Mga_v1-05527;Mga_v1-05528;Mga_v1-05578;Mga_v1-06210;Mga_v1-06264;Mga_v1-06443;Mga_v1-06799;Mga_v1-06996;Mga_v1-07411;Mga_v1-07638;Mga_v1-07915;Mga_v1-07954;Mga_v1-07959;Mga_v1-08178;Mga_v1-08247;Mga_v1-08336;Mga_v1-08554;Mga_v1-08616;Mga_v1-08719;Mga_v1-08773;Mga_v1-08852;Mga_v1-08878;Mga_v1-09086;Mga_v1-09092;Mga_v1-09214;Mga_v1-09249;Mga_v1-09271;Mga_v1-09371;Mga_v1-09612;Mga_v1-09613;Mga_v1-09775;Mga_v1-09839;Mga_v1-09894;Mga_v1-09904;Mga_v1-09990;Mga_v1-10081;Mga_v1-10118;Mga_v1-10129;Mga_v1-10365;Mga_v1-10629;Mga_v1-10650;Mga_v1-10792;Mga_v1-10829
Pyrimidine Nucleobase Metabolic Process (GO:0006206)	25	Mga_v1-00096;Mga_v1-00129;Mga_v1-00203;Mga_v1-01421;Mga_v1-02576;Mga_v1-02888;Mga_v1-03017;Mga_v1-03615;Mga_v1-04765;Mga_v1-05250;Mga_v1-05634;Mga_v1-

		06237;Mga_v1-06999;Mga_v1-07079;Mga_v1-07311;Mga_v1-08198;Mga_v1-08741;Mga_v1-09243;Mga_v1-09279;Mga_v1-09633;Mga_v1-10090;Mga_v1-10259;Mga_v1-10374;Mga_v1-10472;Mga_v1-10585
Electron Transport (GO:0006118)	23	Mga_v1-00529;Mga_v1-01486;Mga_v1-01789;Mga_v1-02856;Mga_v1-03356;Mga_v1-03615;Mga_v1-03784;Mga_v1-04088;Mga_v1-04680;Mga_v1-04803;Mga_v1-04841;Mga_v1-05250;Mga_v1-05865;Mga_v1-07181;Mga_v1-07643;Mga_v1-09243;Mga_v1-09412;Mga_v1-09632;Mga_v1-09633;Mga_v1-09682;Mga_v1-09865;Mga_v1-10148;Mga_v1-10568
Dicarboxylic Acid Biosynthetic Process (GO:0043650)	13	Mga_v1-01082;Mga_v1-03260;Mga_v1-04088;Mga_v1-05250;Mga_v1-06999;Mga_v1-07430;Mga_v1-07431;Mga_v1-07643;Mga_v1-07995;Mga_v1-08947;Mga_v1-09125;Mga_v1-09632;Mga_v1-09633
Phospholipid Catabolic Process (GO:0009395)	11	Mga_v1-01846;Mga_v1-02157;Mga_v1-02890;Mga_v1-05345;Mga_v1-05925;Mga_v1-06345;Mga_v1-08403;Mga_v1-09379;Mga_v1-09469;Mga_v1-10586;Mga_v1-10887
Glutamate Biosynthetic Process (GO:0006537)	10	Mga_v1-01082;Mga_v1-04088;Mga_v1-05250;Mga_v1-07430;Mga_v1-07431;Mga_v1-07643;Mga_v1-08947;Mga_v1-09125;Mga_v1-09632;Mga_v1-09633
Peroxidase Reaction (GO:0006804)	9	Mga_v1-02221;Mga_v1-03897;Mga_v1-06585;Mga_v1-06789;Mga_v1-06949;Mga_v1-07653;Mga_v1-08923;Mga_v1-09253;Mga_v1-

		09297
Panthotenate Biosynthetic Process (GO:0015940)	7	Mga_v1-00845;Mga_v1-03615;Mga_v1-05250;Mga_v1-06743;Mga_v1-08075;Mga_v1-09243;Mga_v1-09633
