# Supplementary material for: A First Insight into the Genome of the Filter-Feeder Mussel Mytilus galloprovincialis
Source: PLoS One. 2016 Mar 15;11(3):e0151561. doi: 10.1371/journal.pone.0151561 (PMC4792442; doi:10.1371/journal.pone.0151561)
Supplement: S1 Table — The table shows the number of variants present in the mitochondrial genes. The following information is given: gene name, size of the gene in base-pairs, absolute number of variants found and percentage of variants related to the gene length. (PDF) [file pone.0151561.s004.pdf]

S1 Table: Variants in Mitochondrial Genes

KM192128 (F)			
gene	#	length (bp)	%
**ND4**	34	1308	2.599
**ND6**	12	465	2.581
**COX3**	19	793	2.396
**ND3**	8	351	2.279
**ND2**	17	948	1.793
**CYTB**	21	1308	1.606
**ND5**	26	1728	1.505
**COX1**	24	1656	1.449
**ATP8**	3	255	1.176
**ATP6**	8	717	1.116
**ND4L**	3	282	1.064
**COX2**	7	729	0.960
**ND1**	10	1079	0.927

KM192129 (M)			
gene	#	length (bp)	%
**CYTB**	21	1311	1.602
**ND4L**	4	282	1.418
**ATP6**	10	717	1.395
**COX1**	23	1740	1.322
**ND4**	16	1308	1.223
**ND5**	21	1725	1.217
**COX2**	8	729	1.097
**ATP8**	3	330	0.909
**ND6**	4	465	0.860
**ND3**	3	351	0.855
**ND2**	8	948	0.844
**ND1**	8	1067	0.750
**COX3**	6	936	0.641
