# Supplementary material for: A First Insight into the Genome of the Filter-Feeder Mussel Mytilus galloprovincialis
Source: PLoS One. 2016 Mar 15;11(3):e0151561. doi: 10.1371/journal.pone.0151561 (PMC4792442; doi:10.1371/journal.pone.0151561)
Supplement: S2 Table — The following information is given: GO-ID, GO-term, Number of proteins in the organism for that GO annotation, False Discovery Rate value, P-value and whether it is up or downregulated. For those non-significant terms, the symbol ⌘ is used. When significant, “++” is used for proteins overrepresented in M. galloprovincialis, and “–-” for those underrepresented. (PDF) [file pone.0151561.s005.pdf]

S2 Table

GO ID	GO Term	*M. galloprovincialis*	*P. fucata*				*C. gigas*				*L. gigantea*				*A. californica*			
		N seqs	N seqs	FDR	P-Value	Up/Down	N Seqs	FDR	P-Value	Up/Down	N Seqs	FDR	P-Value	Up/Down	N Seqs	FDR	P-Value	Up/Down
GO:0002253	activation of immune response	4	31	⌘			25	⌘			60	3.778E-03	1.297E-04	--	38	⌘		
GO:0042967	acyl-carrier-protein biosynthetic process	18	92	⌘			83	4.313E-01	1.928E-02	--	0	1.515E-09	1.792E-11	++	113	⌘		
GO:0031145	anaphase-promoting complex-dependent proteasomal ubiquitir	0	23	⌘			25	2.203E-02	4.544E-04	--	29	8.830E-03	3.489E-04	--	44	9.993E-04	1.565E-05	--
GO:0048646	anatomical structure formation involved in morphogenesis	65	227	⌘			181	⌘			315	3.454E-03	1.165E-04	--	187	⌘		
GO:0002478	antigen processing and presentation of exogenous peptide antig	0	0	⌘			0	⌘			36	1.716E-03	5.271E-05	--	1	⌘		
GO:0006200	ATP catabolic process	92	149	2.227E-06	4.460E-09	++	66	2.168E-12	3.210E-15	++	123	1.188E-06	1.913E-08	++	74	8.089E-17	8.972E-20	++
GO:0048514	blood vessel morphogenesis	11	78	⌘			58	4.482E-01	2.212E-02	--	101	1.487E-03	4.508E-05	--	56	⌘		
GO:0048593	camera-type eye morphogenesis	2	19	⌘			17	⌘			48	3.454E-03	1.166E-04	--	23	⌘		
GO:0003205	cardiac chamber development	1	8	⌘			7	⌘			39	4.925E-03	1.784E-04	--	11	⌘		
GO:0007050	cell cycle arrest	2	29	⌘			25	3.856E-01	1.648E-02	--	52	1.664E-03	5.085E-05	--	56	3.356E-03	6.361E-05	--
GO:0045165	cell fate commitment	14	63	⌘			65	6.853E-01	4.386E-02	--	143	1.262E-05	2.373E-07	--	76	⌘		
GO:0000902	cell morphogenesis	67	204	⌘			162	⌘			319	4.663E-03	1.678E-04	--	212	⌘		
GO:0000904	cell morphogenesis involved in differentiation	48	146	⌘			122	⌘			229	3.120E-02	1.590E-03	--	155	⌘		
GO:0016044	cellular membrane organization	18	106	⌘			92	1.308E-01	3.989E-03	--	198	1.599E-08	2.010E-10	--	139	1.421E-02	3.625E-04	--
GO:0044267	cellular protein metabolic process	266	1303	2.359E-04	1.446E-06	--	985	1.467E-07	1.404E-09	--	1218	3.612E-11	3.620E-13	--	1297	1.116E-07	1.047E-09	--
GO:0033554	cellular response to stress	90	323	⌘			243	⌘			406	3.353E-03	1.118E-04	--	338	⌘		
GO:0007417	central nervous system development	40	169	⌘			146	6.444E-01	3.975E-02	--	242	2.365E-04	5.633E-06	--	136	⌘		
GO:0043009	chordate embryonic development	26	127	⌘			100	⌘			185	1.846E-04	4.201E-06	--	94	⌘		
GO:0031497	chromatin assembly	2	9	⌘			35	3.500E-02	7.870E-04	--	108	9.060E-10	1.040E-11	--	30	⌘		
GO:0006333	chromatin assembly or disassembly	3	33	⌘			42	3.040E-02	6.690E-04	--	114	1.910E-09	2.310E-11	--	34	⌘		
GO:0016568	chromatin modification	20	159	2.489E-02	3.370E-04	--	114	1.590E-02	3.220E-04	--	184	1.360E-06	2.220E-08	--	138	4.980E-02	1.560E-03	--
GO:0006325	chromatin organization	21	180	4.686E-03	4.261E-05	--	149	⌘			284	5.615E-15	4.151E-17	--	164	3.388E-03	6.558E-05	--
GO:0006325	chromatin organization	21	180	⌘			149	5.630E-05	7.230E-07	--	294	⌘			164	⌘		
GO:0006338	chromatin remodeling	2	39	⌘			24	3.820E-01	1.610E-02	--	32	⌘			23	⌘		
GO:0006613	cotranslational protein targeting to membrane	0	11	⌘			6	⌘			24	2.960E-02	1.490E-03	--	8	⌘		
GO:0016569	covalent chromatin modification	17	112	⌘			91	9.280E-02	2.570E-03	--	154	2.500E-05	4.930E-07	--	112	⌘		
GO:0016482	cytoplasmic transport	34	138	⌘			102	⌘			210	5.376E-04	1.409E-05	--	145	⌘		
GO:0043650	dicarboxylic acid biosynthetic process	13	20	⌘			12	3.262E-01	1.310E-02	++	5	1.322E-03	3.952E-05	++	14	⌘		
GO:0006977	DNA damage response, signal transduction by p53 class mediato	0	14	⌘			8	⌘			21	⌘			33	1.100E-02	2.630E-04	--
GO:0006118	electron transport	23	98	⌘			56	⌘			0	2.078E-12	1.826E-14	++	56	⌘		
GO:0022900	electron transport chain	2	31	⌘			23	4.560E-01	2.440E-02	--	16	⌘			37	⌘		
GO:0048562	embryonic organ morphogenesis	11	60	⌘			70	9.557E-02	2.678E-03	--	113	2.347E-04	5.573E-06	--	74	⌘		
GO:0060562	epithelial tube morphogenesis	11	62	⌘			72	5.672E-02	1.388E-03	--	110	3.116E-04	7.730E-06	--	80	⌘		
GO:0072594	establishment of protein localization to organelle	12	59	⌘			41	⌘			107	1.487E-03	4.477E-05	--	56	⌘		
GO:0038093	Fc receptor signaling pathway	0	0	⌘			0	⌘			39	7.149E-04	1.938E-05	--	0	⌘		
GO:0008543	fibroblast growth factor receptor signaling pathway	2	13	⌘			12	⌘			49	2.377E-03	7.595E-05	--	15	⌘		
GO:0006537	glutamate biosynthetic process	10	5	1.733E-02	2.192E-04	++	2	6.480E-03	1.196E-04	++	4	9.570E-03	3.828E-04	++	3	3.067E-03	5.660E-05	++
GO:0003007	heart morphogenesis	8	50	⌘			43	6.847E-01	4.358E-02	--	97	1.924E-04	4.457E-06	--	62	⌘		
GO:0016573	histone acetylation	3	51	⌘			42	3.038E-02	6.686E-04	--	58	1.487E-03	4.496E-05	--	44	⌘		
GO:0002376	immune system process	55	233	⌘			205	2.550E-01	9.700E-03	--	369	2.050E-08	2.620E-10	--	241	⌘		
GO:0045087	innate immune response	10	55	⌘			53	5.818E-01	3.416E-02	--	118	2.818E-05	5.647E-07	--	76	⌘		
GO:0007243	intracellular protein kinase cascade	26	111	⌘			96	⌘			168	1.810E-03	5.651E-05	--	136	⌘		
GO:0030522	intracellular receptor signaling pathway	2	20	⌘			13	⌘			53	1.069E-03	3.146E-05	--	31	⌘		
GO:0097193	intrinsic apoptotic signaling pathway	3	11	⌘			10	⌘			53	4.447E-03	1.578E-04	--	5	⌘		
GO:0043414	macromolecule methylation	6	66	⌘			38	5.173E-01	2.915E-02	--	74	2.449E-03	7.884E-05	--	55	⌘		
GO:0007018	microtubule-based movement	81	153	5.046E-04	3.890E-06	++	62	3.367E-10	1.462E-12	++	72	2.978E-11	2.910E-13	++	89	2.783E-10	1.123E-12	++
GO:0007017	microtubule-based process	113	244	9.680E-04	8.036E-06	++	146	1.010E-05	1.190E-07	++	302	⌘			211	6.310E-05	8.320E-07	++
GO:0006397	mRNA processing	27	145	⌘			126	9.580E-02	2.720E-03	--	144	⌘			154	⌘		
GO:2001234	negative regulation of apoptotic signaling pathway	0	0	⌘			0	⌘			23	4.790E-02	2.600E-03	--	0	⌘		
GO:0060548	negative regulation of cell death	23	0	⌘			61	⌘			154	2.394E-03	7.670E-05	--	78	⌘		
GO:0045596	negative regulation of cell differentiation	18	69	⌘			68	⌘			132	2.017E-03	6.331E-05	--	76	⌘		
GO:0032269	negative regulation of cellular protein metabolic process	8	74	⌘			68	1.402E-02	2.795E-04	--	90	7.365E-04	2.008E-05	--	93	5.670E-03	1.132E-04	--
GO:0031936	negative regulation of chromatin silencing	3	0	⌘			0	4.480E-01	2.270E-02	++	0	⌘			1	⌘		
GO:0031397	negative regulation of protein ubiquitination	0	23	⌘			22	5.150E-02	1.240E-03	--	29	8.830E-03	3.490E-04	--	42	1.480E-03	2.510E-05	--
GO:0043433	negative regulation of sequence-specific DNA binding transcript	1	14	⌘			11	⌘			29	4.860E-02	2.670E-03	--	12	⌘		
GO:0009968	negative regulation of signal transduction	28	101	⌘			63	⌘			187	4.394E-04	1.123E-05	--	104	⌘		
GO:0000122	negative regulation of transcription from RNA polymerase II pro	5	57	⌘			62	1.992E-03	3.263E-05	--	168	8.747E-14	7.042E-16	--	67	2.229E-02	5.933E-04	--

GO:0045892	negative regulation of transcription, DNA-dependent	15	112	⌘			112	6.828E-04	1.004E-05	--	238	6.151E-14	4.901E-16	--	131	4.985E-03	9.903E-05	--
GO:0051436	negative regulation of ubiquitin-protein ligase activity involved i	0	21	⌘			18	1.109E-01	3.284E-03	--	22	4.475E-02	2.397E-03	--	39	3.376E-03	6.502E-05	--
GO:0050877	neurological system process	61	236	⌘			190	⌘			295	5.340E-03	1.950E-04	--	272	⌘		
GO:0030182	neuron differentiation	79	231	⌘			183	⌘			383	7.050E-04	1.899E-05	--	237	⌘		
GO:0031175	neuron projection development	49	147	⌘			121	⌘			231	3.330E-02	1.720E-03	--	152	⌘		
GO:1990138	neuron projection extension	13	21	⌘			8	8.290E-02	2.260E-03	++	24	⌘			21	⌘		
GO:1902284	neuron projection extension involved in neuron projection guida	8	4	⌘			1	1.390E-02	2.770E-04	++	7	⌘			4	4.880E-02	1.520E-03	++
GO:0031102	neuron projection regeneration	5	7	⌘			3	6.850E-01	4.580E-02	++	4	⌘			4	⌘		
GO:0007218	neuropeptide signaling pathway	0	7	⌘			1	⌘			12	⌘			42	1.479E-03	2.506E-05	--
GO:0007219	Notch signaling pathway	5	28	⌘			21	⌘			65	4.243E-03	1.499E-04	--	27	⌘		
GO:0006334	nucleosome assembly	2	8	⌘			32	7.193E-02	1.888E-03	--	102	5.552E-09	6.841E-11	--	29	⌘		
GO:0034728	nucleosome organization	2	14	⌘			36	3.620E-02	8.260E-04	--	110	5.960E-10	6.650E-12	--	30	⌘		
GO:0072384	organelle transport along microtubule	5	8	⌘			3	6.850E-01	4.580E-02	++	15	⌘			9	⌘		
GO:0015940	pantothenate biosynthetic process	7	7	⌘			5	6.909E-01	4.664E-02	++	0	2.115E-03	6.687E-05	++	5	⌘		
GO:0006804	peroxidase reaction	9	16	⌘			15	⌘			0	1.874E-04	4.280E-06	++	15	⌘		
GO:0009395	phospholipid catabolic process	11	21	⌘			11	5.506E-01	3.184E-02	++	2	4.872E-04	1.261E-05	++	22	⌘		
GO:0071158	positive regulation of cell cycle arrest	0	14	⌘			8	⌘			23	4.790E-02	2.600E-03	--	33	1.100E-02	2.630E-04	--
GO:0090068	positive regulation of cell cycle process	2	21	⌘			16	⌘			54	7.476E-04	2.051E-05	--	46	2.879E-02	8.042E-04	--
GO:0010720	positive regulation of cell development	2	13	⌘			10	⌘			40	2.170E-02	1.020E-03	--	29	⌘		
GO:0045597	positive regulation of cell differentiation	10	55	⌘			37	⌘			106	3.067E-04	7.580E-06	--	56	⌘		
GO:0008284	positive regulation of cell proliferation	13	62	⌘			62	6.296E-01	3.871E-02	--	117	5.907E-04	1.577E-05	--	77	⌘		
GO:0032270	positive regulation of cellular protein metabolic process	20	100	⌘			98	1.640E-01	5.290E-03	--	170	1.740E-05	3.340E-07	--	117	⌘		
GO:0001819	positive regulation of cytokine production	2	13	⌘			8	⌘			38	3.020E-02	1.540E-03	--	10	⌘		
GO:0043547	positive regulation of GTPase activity	14	40	⌘			16	6.440E-01	3.970E-02	++	46	⌘			31	⌘		
GO:0002684	positive regulation of immune system process	9	51	⌘			47	⌘			93	1.060E-03	3.060E-05	--	58	⌘		
GO:0045089	positive regulation of innate immune response	3	22	⌘			24	⌘			47	1.880E-02	8.490E-04	--	38	⌘		
GO:0010740	positive regulation of intracellular protein kinase cascade	10	42	⌘			36	⌘			82	1.570E-02	6.950E-04	--	71	⌘		
GO:0044093	positive regulation of molecular function	39	161	⌘			138	⌘			228	8.353E-04	2.347E-05	--	192	⌘		
GO:0010638	positive regulation of organelle organization	3	25	⌘			28	⌘			66	2.299E-04	5.438E-06	--	35	⌘		
GO:0010638	positive regulation of organelle organization	3	25	⌘			28	4.640E-01	2.570E-02	--	66	⌘			35	⌘		
GO:0045937	positive regulation of phosphate metabolic process	14	63	⌘			57	⌘			120	7.801E-04	2.160E-05	--	69	⌘		
GO:0031401	positive regulation of protein modification process	14	87	⌘			86	4.616E-02	1.087E-03	--	153	1.630E-06	2.665E-08	--	98	⌘		
GO:0031398	positive regulation of protein ubiquitination	1	27	⌘			31	2.590E-02	5.480E-04	--	38	7.440E-03	2.870E-04	--	49	3.190E-03	5.980E-05	--
GO:0032320	positive regulation of Ras GTPase activity	8	19	⌘			6	5.490E-01	3.160E-02	++	31	⌘			11	⌘		
GO:0009967	positive regulation of signal transduction	28	89	⌘			77	⌘			171	3.975E-03	1.391E-04	--	117	⌘		
GO:1901798	positive regulation of signal transduction by p53 class mediator	5	2	⌘			1	2.230E-01	8.330E-03	++	0	2.170E-02	1.040E-03	++	0	2.320E-02	6.330E-04	++
GO:0045944	positive regulation of transcription from RNA polymerase II pror	6	91	5.746E-03	5.475E-05	--	79	1.051E-04	1.431E-06	--	209	1.936E-17	1.050E-19	--	106	5.313E-05	6.803E-07	--
GO:0045893	positive regulation of transcription, DNA-dependent	17	150	1.176E-02	1.360E-04	--	132	6.320E-05	8.360E-07	--	289	7.470E-18	3.990E-20	--	154	9.000E-04	1.370E-05	--
GO:0051437	positive regulation of ubiquitin-protein ligase activity involved ir	0	20	⌘			22	5.151E-02	1.240E-03	--	23	4.789E-02	2.604E-03	--	41	2.261E-03	4.127E-05	--
GO:0043687	post-translational protein modification	6	39	⌘			40	4.480E-01	2.090E-02	--	20	⌘			52	⌘		
GO:0043161	proteasomal ubiquitin-dependent protein catabolic process	5	58	⌘			49	5.068E-02	1.200E-03	--	73	7.595E-04	2.090E-05	--	83	1.176E-03	1.885E-05	--
GO:0006486	protein glycosylation	5	62	⌘			30	⌘			67	3.093E-03	1.024E-04	--	49	⌘		
GO:0051258	protein polymerization	12	52	⌘			41	⌘			127	3.166E-05	6.449E-07	--	57	⌘		
GO:0006605	protein targeting	12	73	⌘			46	⌘			108	1.069E-03	3.152E-05	--	62	⌘		
GO:0006612	protein targeting to membrane	1	16	⌘			7	⌘			32	2.260E-02	1.100E-03	--	10	⌘		
GO:0071824	protein-DNA complex subunit organization	2	21	⌘			45	3.223E-03	5.566E-05	--	120	⌘			40	⌘		
GO:0071824	protein-DNA complex subunit organization	2	21	⌘			45	⌘			120	4.070E-11	4.150E-13	--	40	⌘		
GO:0006144	purine nucleobase metabolic process	34	143	⌘			81	⌘			15	8.989E-09	1.115E-10	++	116	⌘		
GO:0006206	pyrimidine nucleobase metabolic process	25	67	⌘			52	⌘			9	3.333E-07	5.038E-09	++	77	⌘		
GO:2001233	regulation of apoptotic signaling pathway	1	0	⌘			0	⌘			49	4.489E-04	1.150E-05	--	1	⌘		
GO:0065008	regulation of biological quality	151	495	⌘			428	⌘			636	8.879E-04	2.502E-05	--	552	⌘		
GO:0071156	regulation of cell cycle arrest	0	17	⌘			9	⌘			28	1.420E-02	6.130E-04	--	36	8.270E-03	1.770E-04	--
GO:0060284	regulation of cell development	26	95	⌘			77	⌘			168	1.810E-03	5.651E-05	--	115	⌘		
GO:0044087	regulation of cellular component biogenesis	12	53	⌘			43	⌘			99	4.713E-03	1.700E-04	--	53	⌘		
GO:0060341	regulation of cellular localization	22	73	⌘			67	⌘			146	3.334E-03	1.109E-04	--	83	⌘		
GO:0032268	regulation of cellular protein metabolic process	51	291	⌘			243	8.360E-04	1.290E-05	--	316	3.670E-06	6.470E-08	--	311	1.500E-03	2.570E-05	--
GO:1902275	regulation of chromatin organization	0	9	⌘			10	⌘			32	3.781E-03	1.307E-04	--	7	⌘		
GO:0031935	regulation of chromatin silencing	3	0	⌘			0	4.480E-01	2.270E-02	++	1	⌘			1	⌘		
GO:0001817	regulation of cytokine production	4	28	⌘			24	⌘			70	4.386E-04	1.117E-05	--	50	⌘		
GO:0035303	regulation of dephosphorylation	0	7	⌘			7	⌘			33	4.127E-03	1.454E-04	--	4	⌘		
GO:0043087	regulation of GTPase activity	40	109	⌘			56	1.780E-01	5.910E-03	++	70	⌘			81	⌘		
GO:0002682	regulation of immune system process	25	84	⌘			81	⌘			172	5.550E-04	1.460E-05	--	85	⌘		
GO:0060080	regulation of inhibitory postsynaptic membrane potential	3	4	⌘			0	4.480E-01	2.270E-02	++	1	⌘			0	⌘		

GO:0010627	regulation of intracellular protein kinase cascade	21	85	⌘			68	⌘			137	7.540E-03	2.920E-04	--	102	⌘		
GO:2000026	regulation of multicellular organismal development	52	185	⌘			159	⌘			307	2.501E-05	4.951E-07	--	194	⌘		
GO:0031396	regulation of protein ubiquitination	1	34	⌘			39	3.172E-03	5.446E-05	--	52	1.997E-04	4.643E-06	--	61	1.853E-04	2.597E-06	--
GO:0051090	regulation of sequence-specific DNA binding transcription factor	3	24	⌘			31	2.953E-01	1.166E-02	--	76	3.242E-05	6.631E-07	--	64	3.146E-03	5.870E-05	--
GO:0044057	regulation of system process	16	64	⌘			58	⌘			103	3.830E-02	1.990E-03	--	56	⌘		
GO:0006355	regulation of transcription, DNA-dependent	80	538	8.157E-06	2.257E-08	--	459	1.694E-11	4.681E-14	--	748	4.063E-29	5.339E-32	--	579	1.582E-10	6.060E-13	--
GO:0006417	regulation of translation	16	116	⌘			84	1.590E-01	5.080E-03	--	55	⌘			116	⌘		
GO:0006446	regulation of translational initiation	7	60	⌘			45	4.330E-01	1.940E-02	--	18	⌘			59	⌘		
GO:0022613	ribonucleoprotein complex biogenesis	25	151	⌘			144	2.643E-03	4.434E-05	--	91	⌘			182	4.142E-03	8.102E-05	--
GO:0006396	RNA processing	46	252	⌘			225	9.475E-04	1.468E-05	--	233	1.124E-02	4.646E-04	--	289	1.377E-03	2.276E-05	--
GO:0009069	serine family amino acid metabolic process	60	238	⌘			175	⌘			22	1.970E-17	1.115E-19	++	240	⌘		
GO:0044341	sodium-dependent phosphate transport	1	5	⌘			0	⌘			41	3.389E-03	1.136E-04	--	1	⌘		
GO:0006614	SRP-dependent cotranslational protein targeting to membrane	0	10	⌘			4	⌘			23	4.790E-02	2.600E-03	--	6	⌘		
GO:0003008	system process	80	295	⌘			249	⌘			402	1.346E-04	2.952E-06	--	333	⌘		
GO:0006367	transcription initiation from RNA polymerase II promoter	0	30	⌘			23	3.235E-02	7.183E-04	--	42	2.944E-04	7.232E-06	--	28	3.734E-02	1.088E-03	--
GO:0006412	translation	41	277	7.634E-03	7.696E-05	--	237	6.637E-06	7.664E-08	--	219	5.895E-03	2.189E-04	--	305	1.069E-05	1.229E-07	--
GO:0006413	translational initiation	8	63	⌘			48	4.000E-01	1.720E-02	--	72	2.230E-02	1.080E-03	--	63	⌘		
GO:0006418	tRNA aminoacylation for protein translation	6	56	⌘			40	4.480E-01	2.090E-02	--	27	⌘			52	⌘		
GO:0006568	tryptophan metabolic process	13	42	⌘			35	⌘			3	1.904E-04	4.394E-06	++	33	⌘		
GO:0016032	viral process	12	30	⌘			68	2.230E-01	8.340E-03	--	110	8.025E-04	2.235E-05	--	99	4.581E-02	1.395E-03	--
